# The effect of menopausal hormone therapy on gastrointestinal cancer risk and mortality in South Korea: a population-based cohort study

**DOI:** 10.1186/s12876-021-02021-y

**Published:** 2021-11-23

**Authors:** Ji Hyung Nam, Sung-In Jang, Hyun Soo Park, Jae Hak Kim, Jun Kyu Lee, Yun Jeong Lim, Moon-Soo Koh, Jin Ho Lee, Sohee Park, Chung Mo Nam, Eun-Cheol Park

**Affiliations:** 1 Division of Gastroenterology, Department of Internal Medicine, Dongguk University Ilsan Hospital, Dongguk University College of Medicine, Goyang, Republic of Korea; 2 Department of Preventive Medicine, Institute of Health Services Research, Yonsei University College of Medicine, 50-1 Yonsei-ro, Seodaemun-gu, Seoul, 03722 Republic of Korea; 3 Department of Obstetrics and Gynecology, Dongguk University Ilsan Hospital, Dongguk University College of Medicine, Goyang, Republic of Korea; 4 Department of Biostatistics, Graduate School of Public Health, Yonsei University, Seoul, Republic of Korea

**Keywords:** Menopausal hormone therapy, Gastrointestinal cancer, Cohort study, Mortality, Dose–response relationship

## Abstract

**Background:**

The effect of menopausal hormone therapy (MHT) on gastrointestinal (GI) cancers is controversial, and no research has been conducted in the East. This study investigates the association between MHT and GI cancer risks in South Korea.

**Methods:**

A prescription-based cohort study was conducted using the NHIS Sample Cohort (2002–2013) of Korea. We used 1:5 propensity score matching, and 22,577 MHT users and 111,113 non-users were selected. Kaplan–Meier survival curves with log-rank tests were used. Cox proportional hazard models were used to estimate hazard ratios (HR) with 95% confidence intervals (CI). Landmark analysis was used to determine dose–response relationship.

**Results:**

The median follow-up was 79.6 of months. Kaplan–Meier survival curve showed less frequent GI cancer diagnoses in MHT users compared to non-users (0.13 vs. 0.16 per 100,000 person-years). Menopausal hormone therapy was associated with decreased incidence of GI cancer (HR = 0.809, 95%CI = 0.691–0.946) and colorectal cancer (CRC) (HR = 0.757, 95%CI = 0.577–0.995). Gastric cancer (GC) incidence showed marginal significance (HR = 0.787, 95%CI = 0.605–1.023). The mortality from GI cancer was lower in MHT users than in non-users (HR = 0.737, 95%CI = 0.547–0.993). The relationship between MHT and GI cancer was stronger with increasing MHT dose in terms of both incidence (*P*_trend_ = 0.0002) and mortality (*P*_trend_ = 0.0064).

**Conclusions:**

The association between MHT use and reduced risks of GI cancers was attributed to CRC and GC and showed a dose–response relationship in a population-based cohort study.

**Supplementary Information:**

The online version contains supplementary material available at 10.1186/s12876-021-02021-y.

## Background

The incidence of gastrointestinal (GI) cancer has been on the decline or plateaued with national interest in cancer screening and surveillance however, GI cancers are still major causes of cancer incidence and mortality worldwide [1]. As the incidence of GI cancer typically increases with age, it may become a major health issue in today’s aging society. Even though most GI cancers are more prevalent in men than women [1], its prevalence rapidly increases with age, even in women. This suggests that female sex hormones may have a protective influence regarding GI cancer risk. According to recent cancer statistics, in South Korea, among women aged 65 years or older, the first and second most common cancers are colorectal and stomach cancer, respectively [2]. These statistics show GI cancer incidence in postmenopausal Korean women rising rapidly [2].

Menopausal hormone therapy (MHT) has been widely used as a treatment for postmenopausal symptoms and menopause-related disorders such as osteoporosis. However, globally their use has declined over the past decades, following reports of associated increased risk of breast cancer [3]. The estimated rate of MHT use in women aged 45–69 years old was 2–9% in European countries in 2010 [4]. In the same year, the annual statistics of Health Insurance in Korea reported that approximately 4.5% of women older than 50 years used MHT [5], comparable to the rate of Western statistics [4].

Several studies reported that exogenous female hormones play a role in intestinal carcinogenesis [6, 7]. Thus, MHT may reduce the incidence of GI cancers, of which the increase in incidence with age precipitates after menopause. Since randomized controlled trials (RCT) showed that MHT reduces the risk of colorectal cancer (CRC) [8, 9], additional RCTs and many observational studies supported the protective effect of MHT on CRC [10–13]. Thus, the evidence from clinical studies may be conclusive, even though some studies exhibited no risk reduction of CRC associated with MHT [14–16]. However, for GI cancer except CRC, there are insufficient studies to prove the association with MHT. In addition, few studies have evaluated GI cancer risk under MHT regimens, except for CRC [14, 15, 17]. Recent studies reported that MHT also reduces the risk of gastric cancer (GC) [17, 18]. However, the number of studies on GC risk associated with MHT is lower than CRC probably because of the low incidence of GC in the West. There are few studies covering the entire spectrum of GI cancers [19]. Moreover, almost all the research so far has been conducted in the West. No population-based studies have been conducted in the East, where the characteristics of the population, the risk factors, and the cancer prevalence may be different from the West. The prevalence of GC is high in South Korea, and CRC is currently increasing [1, 20]. Thus, we planned a nationwide prescription-based cohort study, using National Health Insurance Service (NHIS) Sample Cohort database in South Korea. The aim of the study was to determine the association between MHT use and GI cancer risks, to identify how they differ by MHT regimen or baseline characteristics, and whether there is a dose–response relationship.

## Methods

### Study population

**Fig. 1:**
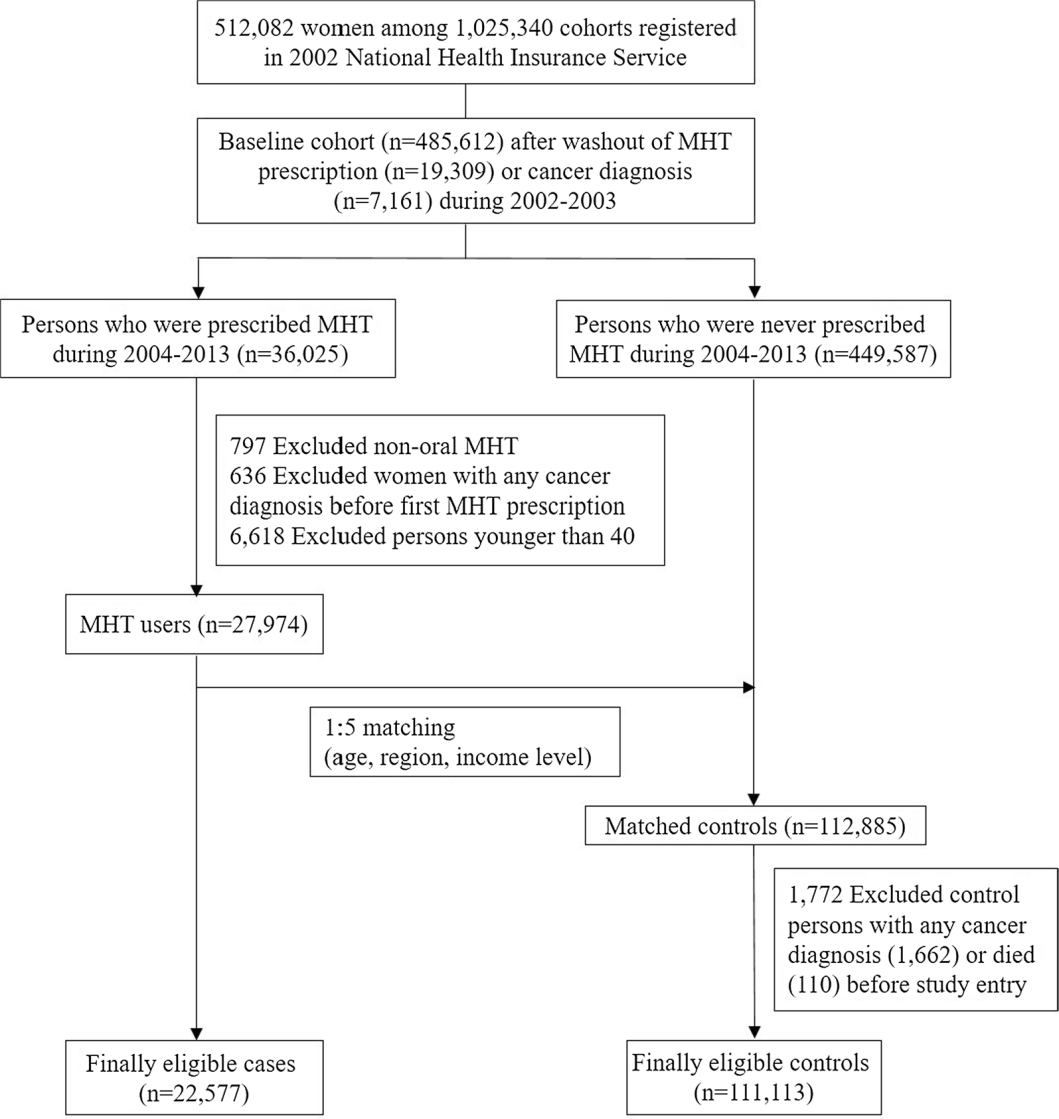
Study flow. MHT, menopausal hormone therapy

The data used in the study were from the NHIS Sample Cohort (2002–2013) of South Korea, a sample of approximately 1,000,000 individuals representative of the general population. The database provides individual patient information such as age, sex, disability or death-related data, residence, income level, insurance coverage, and all medical claim data since 2002. Among the 512,082 women who were registered in the National Sample Cohort in 2002, we first excluded patients who were prescribed MHT or those who visited a hospital with any cancers between 2002 and 2003 in order to washout previous MHT use or history of cancer diagnosis before 2004 (Fig. 1). A diagnosis of any cancer was determined by the International Classification of Diseases 10th (ICD-10) C code. Using the baseline cohort (n = 485,612) after a two-year (2002, 2003) washout, we identified 36,025 MHT users during 2004–2013 and 449,587 persons who never prescribed MHT. We included only MHT regimens with oral administration. The date of cohort entry in the MHT users was determined as the first date of MHT prescription. After excluding MHT users who were diagnosed with any cancers or died before the date of cohort entry and MHT users younger than 40 years old, we selected 27,974 MHT users during 2004–2013. To design the matched cohort study using the MHT users as the case group and to reduce bias, we established the control group by propensity score matching. Propensity scores to estimate the probability of receiving MHT were created using logistic regression. We used a 1:5 case–control matched analysis to select MHT non-users. Covariates for the propensity score model included age (5-year intervals), region (metropolitan or other region), and income level (four categories). The algorithm used greedy nearest neighbor matching without replacement. We selected sequentially treated subjects in the order of the best possible matches, which are those with the highest digit match on propensity score [21]. The income level was determined based on the insurance coverage of claim data. The entire South Korea population is mandated to enroll in National Health Insurance and pay insurance premium based on their salary or property. The income level was classified as less than 30% (low income), 31–60%, 61–90%, and more than 91% (high income).

Using propensity score matching, 22,577 MHT users and 112,885 non-users were matched. The date of cohort entry in the non-users was set to the same date of first MHT prescription for each matching case. In the matched controls, the patients who were diagnosed with any cancers or died before the date of cohort entry were additionally excluded. Finally, 111,113 non-users were eligible. ‘Event’ was defined as the development of cancer diagnosis and death from cancer. ‘Censored' was defined as death before the ‘Event’ or when the end of the study (December 31, 2013) was reached. The study period was from the date of cohort entry to ‘Event’ or ‘Censored’ for both MHT users and matched non-users. The study was conducted in accordance with the guidelines of the Declaration of Helsinki and approved by the Institutional Review Board of Dongguk University Ilsan Hospital (IRB no. DUIH 2018–09-010). Because this study was analyzed using NHIS secondary data, informed consent was waived from IRB.

### Variables

The main outcome variable was diagnosis of GI cancer. A diagnosis of cancer was determined by ICD-10 code in the medical statement of claim data. Gastrointestinal cancers included esophageal (C15), stomach (C16), small intestinal (C17), colorectal (C18–C20), liver, gall bladder, biliary duct (C22–C24), and pancreatic (C25) cancer. Secondary outcomes were a diagnosis of any type of cancer, all-cause mortality, and death from any type of cancer or GI cancer.

The use of MHT, the independent variable of interest, corresponded to Anatomical Therapeutic Chemical (ATC) codes; G03C, G03F, and G03H. The MHT prescription codes included estradiol (E2), conjugated equine estrogen (CEE), and tibolone. Tibolone, a selective estrogen receptor modifier (SERM), is commonly prescribed as MHT for menopausal women with a uterus. Menopausal hormone therapy types were classified as estradiol, conjugated estrogen, tibolone, or mixed-type MHT. We also divided the MHT regimens into single (estradiol or CEE alone) or combination (estradiol plus progesterone or CEE plus progesterone, etc.) regimens. Total MHT doses in each case were calculated using the equivalent dose of estrogen. As the relative potency of estradiol 1.0 mg is equal to that of conjugated estrogen 0.625 mg, this equivalent dose was regarded as a defined daily dose (DDD). Because tibolone does not have a comparable standard for relative potency, a standard dose of 2.5 mg per tablet was considered as a DDD.

Other independent variables included Charlson comorbidity index (CCI) [22], the year of study entry, age (10-year interval), income level, and region. The year of study entry refers to the year corresponding to the date of the cohort entry. The CCI was calculated by scoring the comorbid conditions that could affect patients’ health outcomes, and categorizing into four groups from 1 (low risk) to 4 or more (high risk).

### Statistical analyses

Independent sample *t*-tests or chi-square tests were used to compare baseline characteristics between MHT users and non-users. Cancer incidence and mortality rates were shown at a rate per 100,000 person-years. The association of cancer incidence with MHT use and other covariates was analyzed using chi-square tests. Log-rank test and Kaplan–Meier survival curve were used to compare cancer incidence between MHT users and non-users. We performed a survival analysis using Cox proportional hazard model with hazard ratio (HR) and 95% confidence interval (CI) to identify the association of MHT use with GI cancers. The multivariate model included age group, income, region, CCI, and year of study entry. We performed subgroup analyses according to age, income level, region, and CCI score to identify how the HRs from cox models are different by baseline characteristics. All-cause mortality and cancer-related mortality were also compared between MHT users and non-users using the survival analyses. For the survival analyses, MHT use was categorized by types (E2, CEE, and tibolone), single or combination, and total dose. We used Landmark analysis [23] for the dose–response relationship of MHT with cancers, and *P* values for trend was identified. Landmark time point was set 2 years after the first MHT prescription date. Subjects who were diagnosed with any cancers or died within the time point were excluded. Total MHT dose was calculated within the time point, only. In addition, the Landmark data set was used for sensitivity analysis in terms of the relationship between MHT use and GI cancer. *P* values less than 0.05 were considered statistically significant. All statistical analyses were performed using SAS statistical software version 9.4 (Cary, NC).

## Results

### Baseline characteristics

There were 133,690 (831,311 person-years) women in the study. The median follow-up time (Q1, Q3) was 79.6 months (45.8, 106.5); 79.3 months (45.5, 106.5) in MHT users and 79.6 (45.9, 106.6) in non-users. The mean follow-up time (standard deviation) was 74.6 months (36.8), which was not different between MHT users and non-users (*P* = 0.2916, *P*_equality of variances_ = 0.4020). There was no significant difference between MHT users and non-users in terms of age, income level, region, and year of study entry (Table 1). Charlson comorbidity index was higher in non-users than MHT users (*P* < 0.0001).

**Table 1:** Comparison between MHT users and non-users

Characteristic	Total (n = 133,690)		MHT				*P*
			Yes (n = 22,577)		No (n = 111,113)		
Follow-up months, mean (SD)	74.6 (36.8)		74.4 (36.9)		74.7 (36.7)		0.2916
Age (years)	n	%	n	%	n	%	0.9745
40–49	65,008	48.63	10,959	48.54	54,049	48.64	
50–59	50,866	38.05	8590	38.05	42,276	38.05	
60–69	12,902	9.65	2189	9.70	10,713	9.64	
70~	4914	3.68	839	3.72	4075	3.67	
Income level							0.4772
≤ 30% (low)	38,082	28.49	6425	28.46	31,657	28.49	
31–60%	38,390	28.72	6575	29.12	31,815	28.63	
61–90%	33,180	24.82	5555	24.60	27,625	24.86	
≥ 91% (high)	24,038	17.98	4022	17.81	20,016	18.01	
Region							0.7807
Metropolitan	63,621	47.59	10,725	47.50	52,896	47.61	
Others	70,069	52.41	11,852	52.50	58,217	52.39	
CCI							< .0001
1	45,068	33.71	8040	35.61	37,028	33.32	
2	39,650	29.66	7364	32.62	32,286	29.06	
3	15,894	11.89	2994	13.26	12,900	11.61	
4 or more	33,078	24.74	4179	18.51	28,899	26.01	
Year of study entry							0.9954
2004	19,707	14.74	3293	14.59	16,414	14.77	
2005	18,744	14.02	3142	13.92	15,602	14.04	
2006	16,350	12.23	2750	12.18	13,600	12.24	
2007	17,018	12.73	2871	12.72	14,147	12.73	
2008	14,815	11.08	2507	11.10	12,308	11.08	
2009	10,375	7.76	1754	7.77	8621	7.76	
2010	11,128	8.32	1891	8.38	9237	8.31	
2011	8076	6.04	1374	6.09	6702	6.03	
2012	9489	7.10	1623	7.19	7866	7.08	
2013	7988	5.98	1372	6.08	7866	5.95	

*CCI* Charlson comorbidity index, *MHT* menopausal hormone therapy, *SD* standard deviation

### Cancer incidence: univariate analyses

During the study period, 4756 (0.57 per 100,000 person-years) subjects were diagnosed with any type of cancer, which did not differ between MHT users and non-users (0.60 vs. 0.57,* P* = 0.1699) (Table 2). Gastrointestinal cancers were diagnosed in 1290 (0.155 per 100,000 person-years) subjects during the study period, and it was lower in MHT users than in non-users (0.13 vs. 0.16, *P* = 0.0074). By each GI cancer type, CRC was less frequently diagnosed in MHT users than in non-users (0.04 vs. 0.06, *P* = 0.0480), while GC did not differ by MHT use (0.05 vs. 0.06, *P* = 0.0828). The diagnosis of esophageal, hepatobiliary, and pancreatic cancer was not different between MHT users and non-users. The diagnosis of cancer according to the MHT use and baseline characteristics was described in Additional file 1: Table S1.

**Table 2:** Hazard ratios^a^ for development of cancers: multivariate analyses

Outcome variables	MHT users		Non-users		HR	95% CI	*P*
	n	Rate^b^	n	Rate^b^			
Any cancer	838	0.60	3918	0.57	1.053	0.977, 1.134	0.1800
GI cancer	182	0.13	1108	0.16	0.809	0.691, 0.946	0.0081
Gastric	65	0.05	403	0.06	0.787	0.605, 1.023	0.0733
Colorectal	60	0.04	388	0.06	0.757	0.577, 0.995	0.0457
Hepatobiliary	40	0.03	237	0.03	0.847	0.606, 1.186	0.3336
Pancreatic	16	0.01	68	0.01	1.163	0.674, 2.008	0.5872

Other covariates (age group, income, region, Charlson comorbidity index, and year of study entry) were adjusted in each survival analysis

*CI* confidence interval, *GI* gastrointestinal, *HR* hazard ratio, *MHT* menopausal hormone therapy

^a^Adjusted hazard ratios for mortality in MHT users compared to non-users

^b^The incidence rates were calculated per 100,000 person-years

**Fig. 2:**
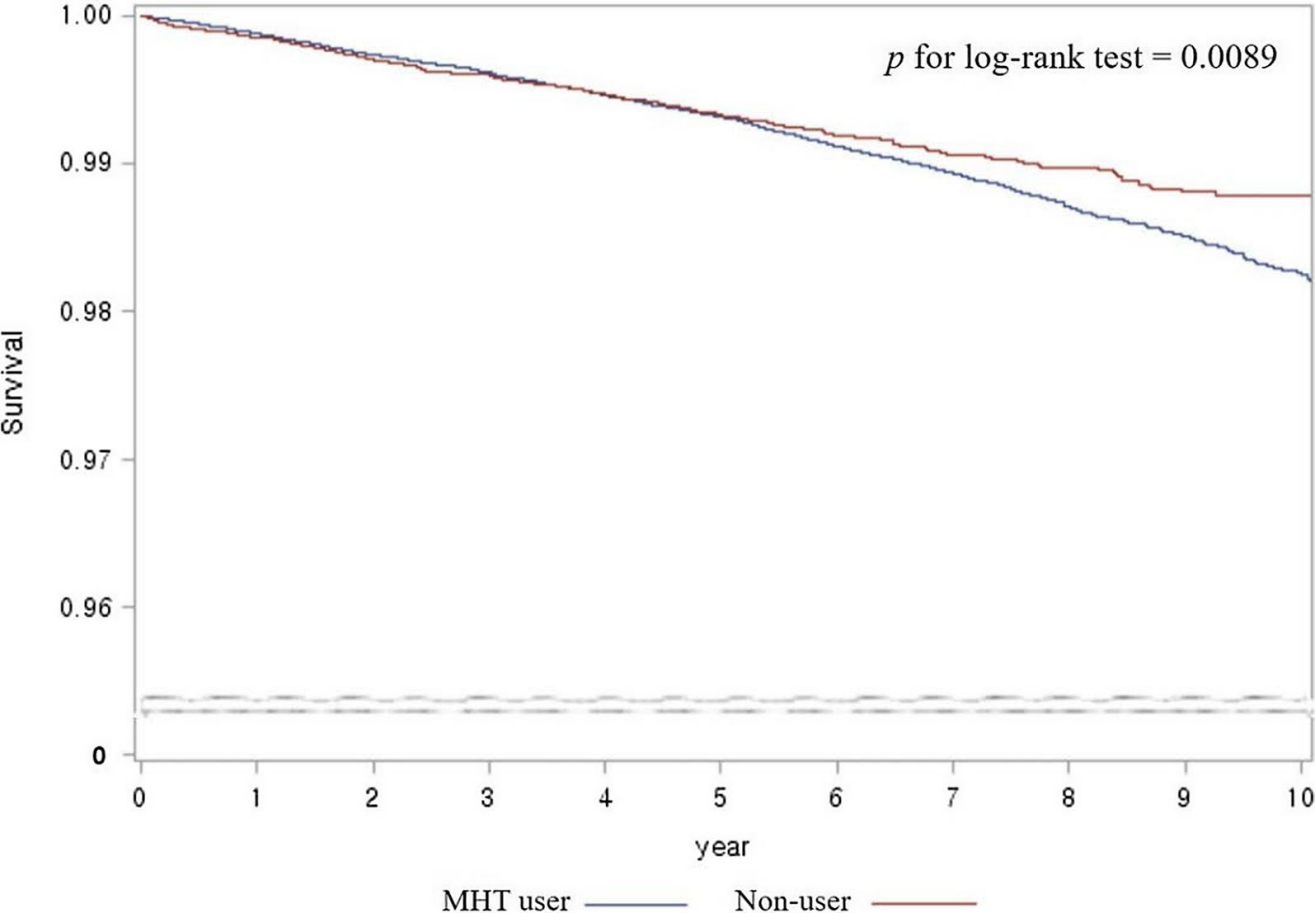
Kaplan–Meier survival curves for gastrointestinal cancer incidence. MHT, menopausal hormone therapy

The Kaplan–Meier survival curves compared the incidence of cancers between MHT users and non-users. Vertical lines indicate survival from diagnosis of cancer, and horizontal lines indicate observation years. Menopausal hormone therapy users were less frequently diagnosed with GI cancer (*P* = 0.0089; Fig. 2). Survival curves show marginal significance in the association of MHT use with GC and CRC (*P* = 0.0887 and *P* = 0.0517, respectively; Additional file 2: Fig. S1). The incidence of hepatobiliary or pancreatic cancer did not differ between MHT users and non-users.

### Cox proportional hazard model: multivariate analyses

Table 2 indicates the survival analyses using the Cox proportional hazard model to determine the relationship between MHT use and the incidence of cancer. Survival analysis for esophageal cancer was not performed because there were too few cases (n = 4). Menopausal hormone therapy was associated with a reduced diagnosis of GI cancer (HR 0.809, 95% CI 0.691–0.946, *P* = 0.0081). By cancer type, MHT use was significantly associated with a reduced diagnosis of CRC (HR 0.757, 95% CI 0.577–0.995, *P* = 0.0457). The association between MHT use and GC diagnosis showed marginal significance (HR 0.787, 95% CI 0.605–1.023, *P* = 0.0733). There was no significant association between MHT use and diagnosis of hepatobiliary or pancreatic cancer (HR: 0.847 and HR: 1.163, respectively).

### Subgroup analyses by MHT regimens and baseline characteristics

We performed survival analyses by MHT regimen (type, combination) using the Cox proportional hazard model (Additional file 3: Table S2). There was no significant difference by MHT type. Meanwhile, MHT with only estrogen was significantly associated with increased incidence of any type of cancer (HR 1.258, *P* = 0.0015). Furthermore, HRs in only estrogen regimen were more than 1.0 in most cancers however, HRs in combination regimen were less than 1.0. Especially, HR for pancreatic cancer diagnosis was significantly increased in MHT users with only estrogen compared to non-users (HR 2.536, *P* = 0.0196).

There were no remarkable differences or tendencies that affected the relationship between MHT use and cancer incidence according to baseline characteristics (Additional file 4: Table S3).

### Menopausal hormone therapy and mortality

Kaplan–Meier survival curves regarding mortality are shown in Additional file 5: Fig. S2. All-cause mortality was lower in MHT users than non-users (*P* < 0.0001), while cancer-related mortality was not significantly associated with MHT. Mortality from GI cancer was lower in MHT users than non-users (*P* = 0.0377). Table 3 shows survival analyses using Cox proportional hazard models to determine the relationship between MHT use and mortality. All-cause mortality was lower in MHT users than in non-users (HR 0.784, *P* < 0.0001), whereas cancer-related mortality was not different between the groups. Also, mortality from GI cancer was lower in MHT users compared to non-users (HR 0.737, *P* = 0.0445), which we attributed to GC and CRC mortality (HR: 0.411 and 0.181, respectively).

**Table 3:** Hazard ratios^a^ for mortality: multivariate analyses

Outcome variables	MHT users		Non-users		HR	95% CI	*P*
	n	Rate^b^	n	Rate^b^			
All-cause mortality	333	0.238	2173	0.314	0.784	0.698, 0.880	< .0001
Cancer-related mortality	115	0.082	661	0.096	0.874	0.717, 1.066	0.1843
Mortality from GI cancer	50	0.036	338	0.049	0.737	0.547, 0.993	0.0445
Gastric	7	0.005	86	0.012	0.411	0.190, 0.890	0.0240
Colorectal	3	0.002	81	0.012	0.181	0.057, 0.572	0.0036
Hepatobiliary	26	0.019	121	0.018	1.077	0.704, 1.647	0.7332
Pancreatic	13	0.009	48	0.007	1.333	0.721, 2.463	0.3598

Other covariates (age group, income, region, Charlson comorbidity index, and year of study entry) were adjusted in each survival analysis

*CI* confidence interval, *GI* gastrointestinal, *HR* hazard ratio, *MHT* menopausal hormone therapy

^a^Adjusted hazard ratios for mortality in MHT users compared to non-users

^b^Mortality rates per 100,000 person-years

### Dose–response relationship

**Fig. 3:**
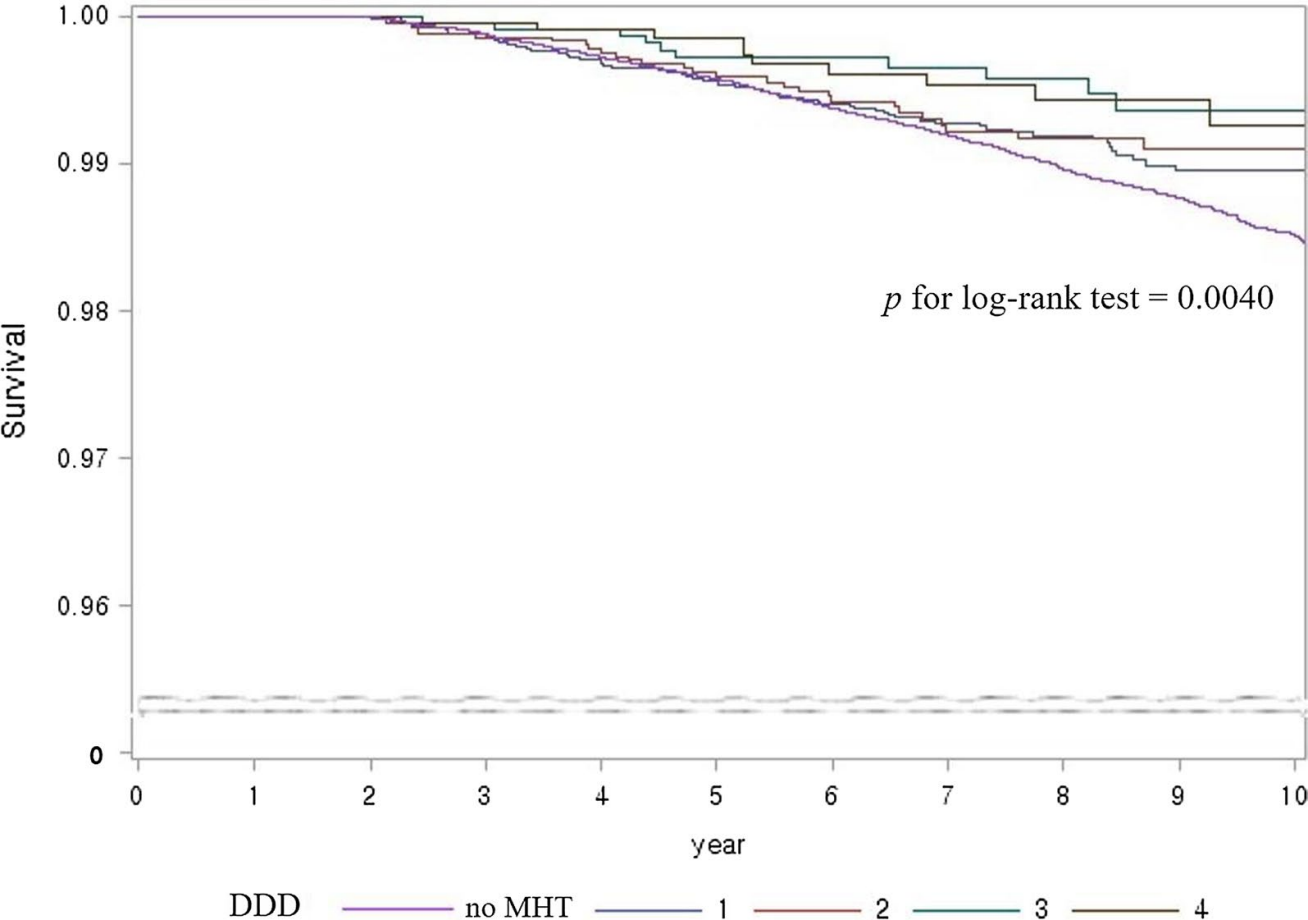
Kaplan–Meier survival curves for gastrointestinal cancer incidence: dose–response relationship. 5 groups were based on DDD of MHT (1, ≤ 100; 2, 100–300; 3, 300–600; 4, ≥ 600; and no MHT). DDD, defined daily dose; MHT, menopausal hormone therapy

We performed Landmark analysis for subgroup by MHT dose, which included 19,543 MHT users and 96,548 non-users. The DDD increased from 1 to 4, with 4 indicating 600 or more of MHT dose. Log rank *P* values by increasing dose for GI cancer incidence was 0.0040 (Fig. 3). The *P* values showed marginal significance in terms of CRC incidence with MHT dose (*P* = 0.0896), and not significant in terms of GC (*P* = 0.2146) (Additional file 6: Fig. S3). In the cox proportional hazard model, the incidence of GI cancer was inversely associated with MHT dose (HR, 0.79, 0.71, 0.49, and 0.55 respectively, *P*_trend_ = 0.0002; Table 4). Also, HRs for CRC diagnosis in MHT users compared to non-users decreased to 0.84, 0.64, 0.63, and 0.15 as MHT dose increased from less than 100 to over 600 of DDD (*P*_trend_ = 0.0069). Hazard ratios for all-cause mortality in MHT users compared to non-users were 0.76, 0.88, 0.64, and 0.34 as MHT dose increased (*P*_trend_ < 0.0001). *P*_trend_ was also significant in cancer-related mortality and GI cancer mortality (0.0052 and 0.0064, respectively), whereas it was not associated with other cancer mortality.

**Table 4:** Hazard ratios for cancer incidence and mortality by MHT dosea: multivariate analyses

	n	Any cancer			GI cancer			Gastric cancer		
		HR	95% CI	*P*	HR	95% CI	*P*	HR	95% CI	*P*
*Incidence*										
DDD no MHT	96,548	1.000	–	0.4709^†^	1.000	–	0.0002^†^	1.000	–	0.0223^†^
≤ 100	10,199	1.004	0.893, 1.128	0.9503	0.786	0.617, 1.002	0.0520	0.790	0.529, 1.180	0.2494
100–300	4423	0.957	0.803, 1.139	0.6189	0.710	0.487, 1.035	0.0746	0.553	0.274, 1.116	0.0982
300–600	2569	1.031	0.827, 1.284	0.7886	0.488	0.269, 0.884	0.0180	0.476	0.178, 1.278	0.1408
≥ 600	2352	0.876	0.681, 1.127	0.3031	0.545	0.292, 1.018	0.0568	0.709	0.293, 1.719	0.4471
*Mortality*										
DDD no MHT	96,548	1.000	–	0.0052^†^	1.000	–	0.0064^†^	1.000	–	0.0264^†^
≤ 100	10,199	0.663	0.470, 0.936	0.0194	0.514	0.300, 0.881	0.0156	0.445	0.139, 1.421	0.1718
100–300	4423	0.891	0.563, 1.410	0.6224	0.798	0.410, 1.553	0.5067	NA^b^	–	–
300–600	2569	0.852	0.455, 1.595	0.6165	0.481	0.154, 1.503	0.2081	NA^b^	–	–
≥ 600	2352	0.113	0.016, 0.803	0.0294	0.213	0.030, 1.521	0.1232	NA^b^	–	–

	n	Colorectal cancer			Hepatobiliary cancer			Pancreas cancer		
		HR	95% CI	*P*	HR	95% CI	*P*	HR	95% CI	*P*
*Incidence*										
DDD no MHT	96,548	1.000		0.0069^†^	1.000		0.1765^†^	1.000		0.6680^†^
≤ 100	10,199	0.843	0.568, 1.251	0.3970	0.639	0.355, 1.148	0.1338	0.825	0.330, 2.065	0.6813
100–300	4423	0.643	0.331, 1.249	0.1926	0.866	0.407, 1.845	0.7095	1.538	0.556, 4.252	0.4065
300–600	2569	0.626	0.258, 1.515	0.2988	0.443	0.110, 1.787	0.2527	NA^c^	-	-
≥ 600	2352	0.152	0.021, 1.082	0.0600	0.874	0.278, 2.743	0.8174	0.904	0.125, 6.558	0.9203
*Mortality*										
DDD no MHT	96,548	1.000		0.0672^†^	1.000		0.3329^†^	1.000		0.8495^†^
≤ 100	10,199	NA^b^	–	–	0.612	0.267, 1.403	0.2464	0.963	0.343, 2.705	0.9429
100–300	4423	NA^b^	–	–	1.476	0.644, 3.380	0.3574	1.666	2.705, 5.411	0.3957
300–600	2569	1.403	0.341, 5.762	0.6387	NA^b^	–	–	NA^b^	–	–
≥ 600	2352	NA^b^	–	–	0.633	0.088, 4.566	0.6505	NA^b^	–	–

Other covariates (age group, income, region, Charlson comorbidity index, and year of study entry) were adjusted in each survival analysis

*CI* confidence interval, *DDD* defined daily dose, *GI* gastrointestinal, *HR* hazard ratio, *MHT* menopausal hormone therapy, *NA* not available

^a^Using Landmark analysis (19,543 MHT users vs. 96,548 non-users)

^b^There was no death from cancer at the MHT dose

^c^There was no cancer diagnosis at the MHT dose

^†^*P*_trend_

### Sensitivity analysis

Next, we performed sensitivity analysis on the main outcomes using the data set included in the Landmark analysis (n = 116,091) (Additional file 7: Table S4). The incidence of GI cancer and CRC was significantly lower in MHT users than in non-users (HR 0.703, 95% CI 0.581–0.852, *P* = 0.0003 and HR 0.693, 95% CI 0.502–0.958, *P* = 0.0266, respectively). These results were comparable to the results of the original data set (n = 133,690). Gastric cancer diagnosis, which showed marginal significance in the original data set, was significantly lower in MHT users compared to non-users (HR 0.684, 95% CI 0.497–0.943, *P* = 0.0202).

## Discussion

We identified that the association between MHT and GI cancer risks using prescription-based women’s sample cohort. This is the first nationwide cohort study conducted in the East and has clinical significance in that it demonstrated a dose–response relationship in the effects of MHT on GI cancer risks. In addition, we performed subgroup analyses according to baseline characteristics and MHT regimens as well as evaluated the effect of MHT on mortality.

The present study included all GI cancers with different pathogenesis. Although we found a reduction in overall GI cancer risk in MHT users, analysis of the effects of MHT on each GI cancer revealed different results for different cancer sites. To summarize, the association between MHT use and reduced risks of GI cancer incidence and mortality was attributed to CRC and GC. Nevertheless, estimates for cancer sites other than CRC are hampered by limited sample size and statistical imprecision and should be validated by further large studies. Statistically, Kaplan–Meier curves for GI cancer incidence crossed between MHT users and non-users. We observed the violation of the proportional hazard assumption by statistical verification, which may imply that MHT needs to be considered as a time-dependent variable. However, as shown in Fig. 2, the curves did not overlap after the middle of follow-up period. Considering that the risk reduction of cancers represents MHT’s long-term effect, the overlap of the curves at the beginning of the follow-up might be ignorable. Moreover, the proportionality assumption was accepted in CRC (*p* = 0.271), the predominant cancer for which risk was decreased by MHT use.

The higher rate of incidence of GI cancer in men than in women and the gradually increasing rate of the incidence after menopause in women suggest a protective role of female hormones against GI cancer. Recent nationwide studies conducted in the West have shown that MHT use lowers the risk of CRC [24–26]. Menopausal hormone therapy was similarly associated with risk reduction of major molecular subtypes of CRC [27]. The effect of MHT on CRC carcinogenesis is known to be related to estrogen receptor beta (ERβ) [6, 7, 28]. High ERβ expression also decreased the risk of morality and cancer recurrence in the CRC patients [29], and a meta-analysis shows that current MHT users have lower risks of colorectal cancer-related and overall mortality [30]. Accordingly, the positive effects of MHT on reducing the risk of CRC seems to be accepted in the literature. However, controversy remains as to whether MHT has a positive effect on CRC prognosis and mortality [31]. It has been asserted that the protective effect of estrogen is limited to the initiation of CRC; once the cancer is developed, estrogen increases proliferation of the disease. This is supported by an RCT that reported a decrease in the risk of CRC that later increased the cumulative hazard of death from CRC in MHT users [8, 32]. If so, women may need a CRC screening before starting MHT. In addition, it is necessary to consider the appropriate duration of MHT in consideration of the various effects of MHT on the progression of intestinal carcinogenesis. We need to perform further research that extends the observation period.

Only nine esophageal cancers were detected during the entire study period. A previous population-based cohort study and a nested case control study found that MHT use is significantly related to decreased esophageal cancer risk, reporting OR = 0.62 and RR = 0.68 respectively [17, 18]. As esophageal cancer is a very rare disease in South Korea, especially among women, it was impossible to evaluate the association with MHT use in this cohort design. Other different approaches are needed in study design and statistical method. Several Western studies showed inconsistent results in the association between GC and MHT use [17, 18, 33]. As estrogen receptor positivity has been reported to be associated with poor outcome in GC patients [34]. Hazard ratio for GC diagnosis in MHT users compared to non-users was 0.787 in our study. It is meaningful because the study was conducted in areas with a high prevalence of GC, but further research is needed to take into account other risk factors such as *Helicobacter pylori*. Hepatobiliary and pancreatic caners are different biologically from other GI cancers that originate in hollow viscus. This may explain why we found that MHT did not affect hepatobiliary or pancreatic cancers. In a recent large prescription-based cohort study in Sweden, MHT significantly decreased the standardized incidence ratio (SIR) of liver cancer compared to the background population, while it was not associated with biliary and pancreatic cancers [19]. An additional matching cohort design using the same data of MHT users found significantly decreased incidence of pancreatic cancer in MHT users compared with matched controls (OR = 0.77) [35]. Our result showed increased HR of pancreatic cancer risk, even though statistical power was insufficient. Especially, the rate of pancreatic cancer diagnosis was significantly increased in the MHT users with only estrogen. Hazard ratio for a diagnosis of any-type cancer was also increased in those with only estrogen. Meanwhile, the median follow-up period was about 6.6 years in our study, and for some cancers it would not have been sufficient to investigate its incidence. As shown in Fig. 2, the survival curves between MHT users and non-users were clearly separated after the middle of the follow-up period. Thus, a study with a longer observation period may allow more favorable results demonstrating MHT’s effect on GI cancer risks.

In addition to the risk of GI cancer, the use of MHT can affect a variety of diseases. Well-known example is the negative aspect of MHT, such as increased risk of breast cancer [36, 37]. Thus, the generalization of our findings to policies that encourage MHT use should be considered with caution. According to previous studies regarding breast cancer and MHT, the longer MHT is used, the greater the risk of breast cancer [38, 39]. However, there is no suggested duration of MHT use without increasing the risk of breast cancer. In our study, GI cancer incidence decreased when using MHT for over two years. Additional studies to evaluate whether the use of MHT for over two years affects breast cancer development would be useful. Meanwhile, a randomized trial showed that early MHT users were significantly less likely than non-users to develop cardiovascular disease (CVD) [40]. They also found decreased mortality related with CVD in MHT users. The lower all-cause mortality rate in MHT users in our study may be related to the effect of MHT on other diseases including CVD. A total duration of MHT use should be determined considering risk-benefits of MHT in the individual.

This study has some limitations. First, we used prescription codes of claim data, thus, the study may not exactly coincide with actual MHT intake in individuals, especially for women who received MHT once or for a short duration. Also, it cannot be asserted that almost all regimens included in the study were prescribed for MHT purposes only, even though the indications include postmenopausal symptoms. Second, for the investigation of rare GI cancers such as esophageal, hepatobiliary, or pancreatic cancer, this study was limited by the small sample size, even though we used a large database. Third, since CCI was initially designed to predict the mortality risk, it should be put into the logistic regression model to calculate the propensity score. Even though we adjusted CCI scores in cox proportional hazard model, the possibility of bias due to confounding effects may still exist. Forth, we used the Kaplan–Meier curves in order to visualize the differences between MHT users and non-users using the cancer diagnosis or mortality as an ‘Event’. However, as the outcome of this study was not related to the absolute cancer risk and survival, the interpretation of the Kaplan–Meier curves may be controversial. Finally, because data relating to cancer stage and treatment was not available in our data, there may be controversy about comparing mortality rates. Further analysis, including data of national cancer registry, is required in the future.

Nevertheless, this is the first nationwide cohort study in Asia to investigate the association between MHT and GI cancer. By using samples from the NHIS claim data for the entire population, study subjects represented the general population of South Korea. Additionally, we analyzed the effects of MHT on mortality as well as incidence of GI cancers and used Landmark analysis to determine the dose–response relationship of MHT with cancer development to minimize the possibility of immortal time bias. For studies on cancer risk, analyses that confirm increasing risk with increasing duration of use are pivotal. To overcome the difficult of determining the exact duration of use with secondary data, we instead analyzed the risk according to defined daily dose (DDD). Since MHT usually has a fixed daily usage, it seems that DDD may represent the duration of use.

## Conclusions

Menopausal hormone therapy was associated with decreased diagnosis of GI cancer, especially for CRC and GC, in Korean women. Furthermore, MHT use was significantly associated with decreased mortality from CRC and GC. These associations of MHT with GI cancer showed a dose–response relationship. This study supports previous researches that found the protective effect of MHT on GI cancers. Our findings, based on national sample cohort data from Korea, warrant the need for long-term follow-up studies.

## Supplementary Information

**Additional file 1. Table S1**. Incidencea of cancers according to MHT use and baseline characteristics.

**Additional file 2. Figure S1**. Kaplan-Meier survival curves for cancer incidence. Vertical lines indicate survival from diagnosis of cancer, and horizontal lines indicate observation years.

**Additional file 3. Table S2**. Hazard ratios for development of cancers: subgroup analyses according to MHT regimens.

**Additional file 4. Table S3**. Hazard ratios^a^ for development of cancers: subgroup analyses according to baseline characteristics.

**Additional file 5. Figure S2**. Kaplan-Meier survival curves for mortality. Vertical lines indicate survival from death, and horizontal lines indicate observation years.

**Additional file 6. Figure S3**. Kaplan-Meier survival curves: dose-response relationship. Vertical linesindicate survival from diagnosis of cancer, and horizontal lines indicate observation years. 5 groups were based on DDD of MHT (1, ≤ 100; 2, 100~300; 3, 300~600; 4, ≥ 600; and no MHT). DDD, defined daily dose; MHT, menopausal hormone therapy.

**Additional file 7. Table S4**. Sensitivity analyses of cancer incidence using Landmark data set (n = 116,091).

## Data Availability

The data in this study available from the corresponding author on reasonable request.

## Abbreviations

ATC: Anatomical Therapeutic Chemical
CCI: Charlson comorbidity index
CEE: Conjugated equine estrogen
CI: Confidence interval
CRC: Colorectal cancer
CVD: Cardiovascular disease
DDD: Defined daily dose
E2: Estradiol
GC: Gastric cancer
GI: Gastrointestinal
HR: Hazard ratio
ICD: International Classification of Diseases
MHT: Menopausal hormone therapy
NHIS: National Health Insurance Service
RCT: Randomized controlled trial
SERM: Selective estrogen receptor modifier
