# Supplementary material for: The effect of menopausal hormone therapy on gastrointestinal cancer risk and mortality in South Korea: a population-based cohort study
Source: BMC Gastroenterol. 2021 Nov 23;21:440. doi: 10.1186/s12876-021-02021-y (PMC8609757; doi:10.1186/s12876-021-02021-y)
Supplement: Supplementary file 1 — Additional file 1. Table S1. Incidencea of cancers according to MHT use and baseline characteristics. [file 12876_2021_2021_MOESM1_ESM.docx]

**The effect of menopausal hormone therapy on gastrointestinal cancer risk and mortality in South Korea: a population-based cohort study**

**Table S1** Incidence^a^ of cancers according to MHT use and baseline characteristics

|  | Total | Any cancer | |  | GI | |  | Esophageal | |  | Gastric | |  | Colorectal | |  | Hepatobiliary | |  | Pancreatic | |  |
| --- | --- | --- | --- | --- | --- | --- | --- | --- | --- | --- | --- | --- | --- | --- | --- | --- | --- | --- | --- | --- | --- | --- |
|  | (person-years) | n | rate |  | n | rate |  | n | rate |  | n | rate |  | N | rate |  | n | rate |  | n | rate |  |
| MHT |  |  |  |  |  |  |  |  |  |  |  |  |  |  |  |  |  |  |  |  |  |  |
| Yes | 139,946 | 838 | 0.60 |  | 182 | 0.13 | ^‡^ | 0 | 0 |  | 65 | 0.05 |  | 60 | 0.04 | ^†^ | 40 | 0.03 |  | 16 | 0.01 |  |
| No | 691,365 | 3,918 | 0.57 |  | 1,108 | 0.16 |  | 4 | 0.0006 |  | 403 | 0.06 |  | 388 | 0.06 |  | 237 | 0.03 |  | 68 | 0.01 |  |
| Age (Years) |  |  |  |  |  |  |  |  |  |  |  |  |  |  |  |  |  |  |  |  |  |  |
| 40-49 | 378,667 | 1,883 | 0.50 | ^§^ | 337 | 0.09 | ^§^ | 0 | 0 |  | 147 | 0.04 | ^§^ | 120 | 0.03 | ^§^ | 50 | 0.01 | ^§^ | 16 | 0.00 | ^§^ |
| 50-59 | 343,559 | 2,070 | 0.60 |  | 583 | 0.17 |  | 3 | 0.0009 |  | 206 | 0.06 |  | 200 | 0.06 |  | 137 | 0.04 |  | 32 | 0.01 |  |
| 60-69 | 82,138 | 544 | 0.66 |  | 227 | 0.28 |  | 1 | 0.0012 |  | 77 | 0.09 |  | 77 | 0.09 |  | 52 | 0.06 |  | 19 | 0.02 |  |
| 70~ | 26,946 | 259 | 0.96 |  | 143 | 0.53 |  | 0 | 0 |  | 38 | 0.14 |  | 51 | 0.19 |  | 38 | 0.14 |  | 17 | 0.06 |  |
| Income level |  |  |  |  |  |  |  |  |  |  |  |  |  |  |  |  |  |  |  |  |  |  |
| ≤30% | 238,266 | 1,527 | 0.64 | ^§^ | 394 | 0.17 |  | 0 | 0 |  | 142 | 0.06 |  | 132 | 0.06 |  | 91 | 0.04 |  | 25 | 0.01 |  |
| 31-60% | 244,018 | 1,318 | 0.54 |  | 388 | 0.16 |  | 2 | 0.0008 |  | 144 | 0.06 |  | 139 | 0.06 |  | 82 | 0.03 |  | 19 | 0.01 |  |
| 61-90% | 206,359 | 1,154 | 0.56 |  | 295 | 0.14 |  | 2 | 0.0010 |  | 105 | 0.05 |  | 102 | 0.05 |  | 60 | 0.03 |  | 23 | 0.01 |  |
| ≥91% | 142,668 | 757 | 0.53 |  | 213 | 0.15 |  | 0 | 0 |  | 77 | 0.05 |  | 75 | 0.05 |  | 44 | 0.03 |  | 17 | 0.01 |  |
| Region |  |  |  |  |  |  |  |  |  |  |  |  |  |  |  |  |  |  |  |  |  |  |
| Metropolitan | 397,177 | 2,398 | 0.60 | ^§^ | 655 | 0.16 | ^†^ | 3 | 0.0008 |  | 233 | 0.06 |  | 238 | 0.06 | ^†^ | 131 | 0.03 |  | 46 | 0.01 |  |
| Others | 434,134 | 2,358 | 0.54 |  | 635 | 0.15 |  | 1 | 0.0002 |  | 235 | 0.05 |  | 210 | 0.05 |  | 146 | 0.03 |  | 38 | 0.01 |  |
| CCI |  |  |  |  |  |  |  |  |  |  |  |  |  |  |  |  |  |  |  |  |  |  |
| 1 | 259,391 | 1291 | 0.50 | ^§^ | 232 | 0.09 | ^§^ | 0 | 0 |  | 103 | 0.04 | ^§^ | 82 | 0.03 | ^§^ | 33 | 0.01 | ^§^ | 10 | 0.00 | ^§^ |
| 2 | 260,853 | 1537 | 0.59 |  | 391 | 0.15 |  | 1 | 0.0004 |  | 153 | 0.06 |  | 135 | 0.05 |  | 79 | 0.03 |  | 21 | 0.01 |  |
| 3 | 105,442 | 682 | 0.65 |  | 240 | 0.23 |  | 2 | 0.0019 |  | 79 | 0.07 |  | 86 | 0.08 |  | 55 | 0.05 |  | 17 | 0.02 |  |
| 4 or more | 205,624 | 1246 | 0.61 |  | 427 | 0.21 |  | 1 | 0.0005 |  | 133 | 0.06 |  | 145 | 0.07 |  | 110 | 0.05 |  | 36 | 0.02 |  |
| Total | 831,311 | 4,756 | 0.57 |  | 1,290 | 0.16 |  | 4 | 0.0005 |  | 468 | 0.06 |  | 448 | 0.05 |  | 277 | 0.03 |  | 84 | 0.01 |  |

CCI, Charlson comorbidity index; GI, gastrointestinal; MHT, menopausal hormone therapy

^a^The incidence rates were calculated per 100,000 person-years

^†^*p*<0.05, ^‡^*p*<0.01, ^§^*p*<0.001
