# Supplementary material for: The effect of menopausal hormone therapy on gastrointestinal cancer risk and mortality in South Korea: a population-based cohort study
Source: BMC Gastroenterol. 2021 Nov 23;21:440. doi: 10.1186/s12876-021-02021-y (PMC8609757; doi:10.1186/s12876-021-02021-y)

**The effect of menopausal hormone therapy on gastrointestinal cancer risk and mortality in South Korea: a population-based cohort study**

**Figure S1**. Kaplan-Meier survival curves for cancer incidence. Vertical lines indicate survival from diagnosis of cancer, and horizontal lines indicate observation years.

(A) Any cancer


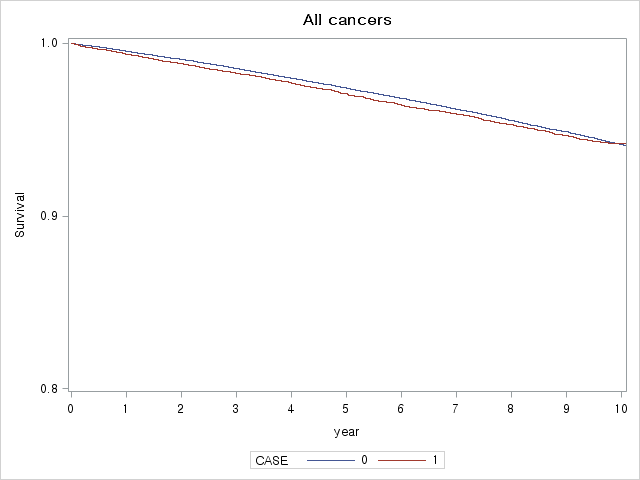


*p* for log-rank test = 0.1467


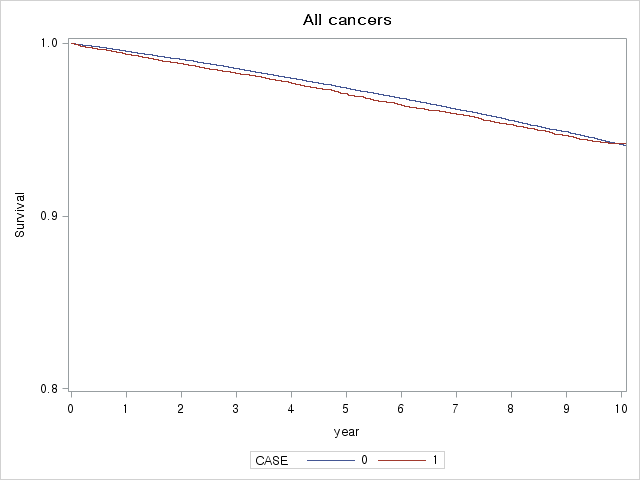

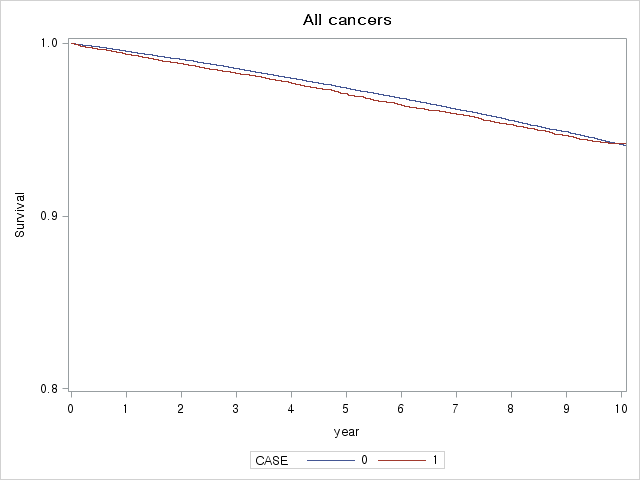

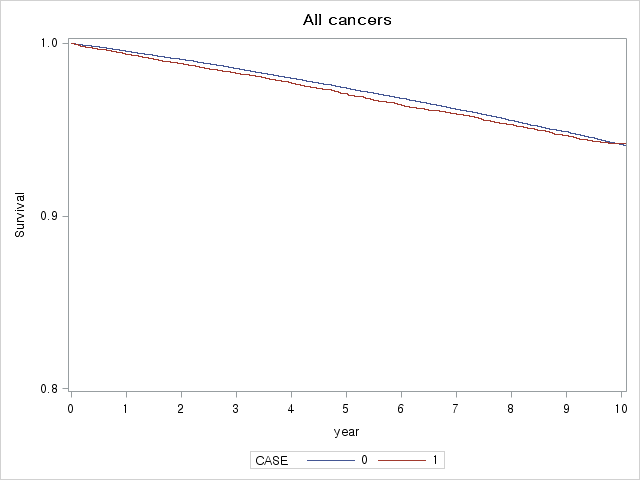


MHT users

Non-users


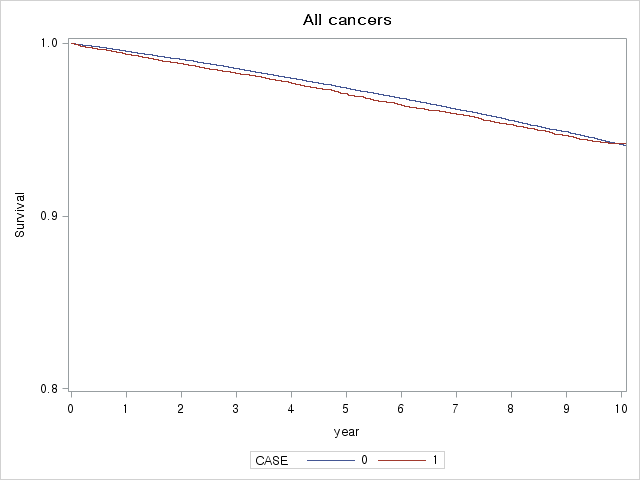


(B) Gastrointestinal cancer


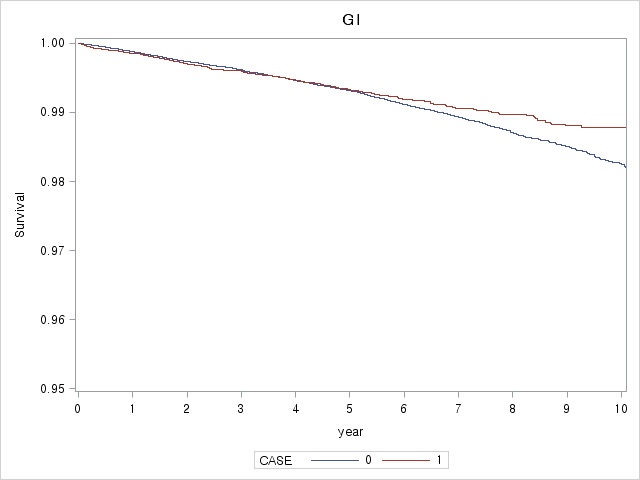


*p* for log-rank test = 0.0089


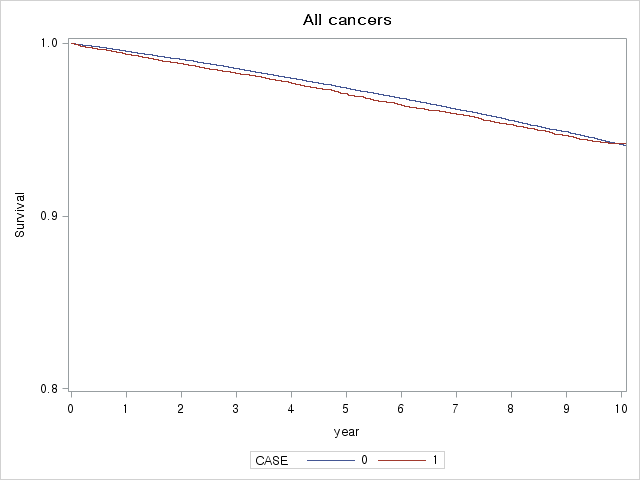

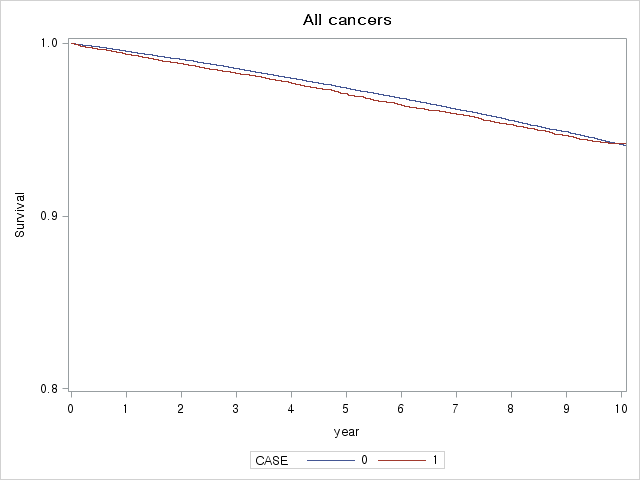

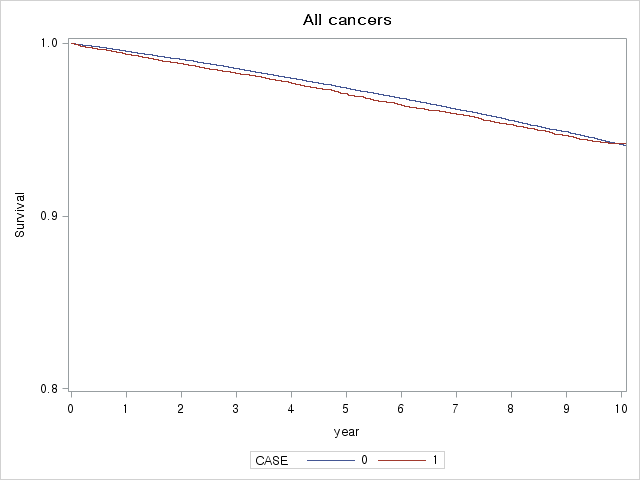


MHT users

Non-users


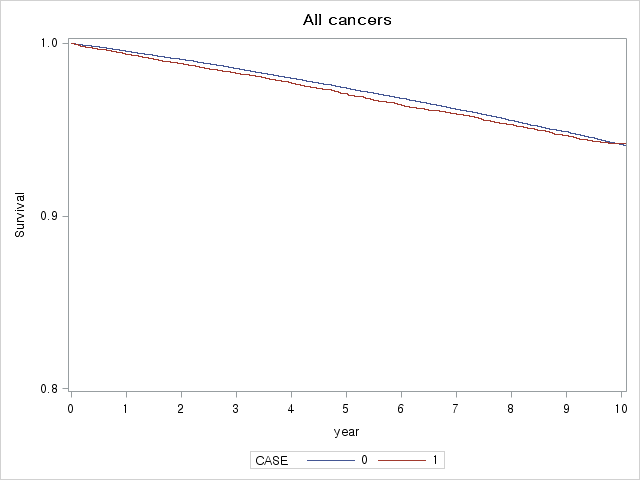


**(C)** Gastric cancer


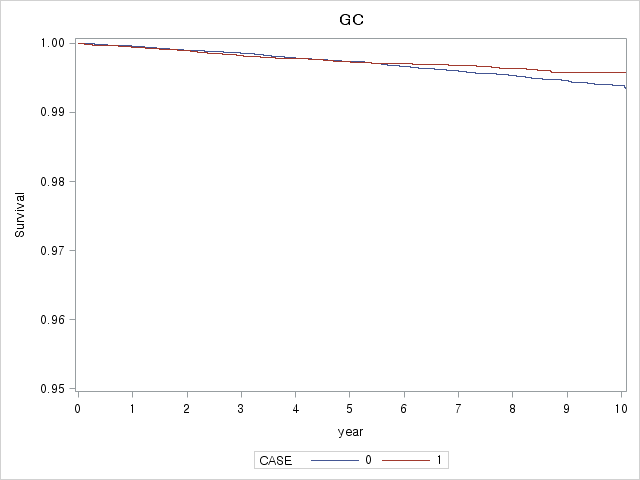


*p* for log-rank test = 0.0887


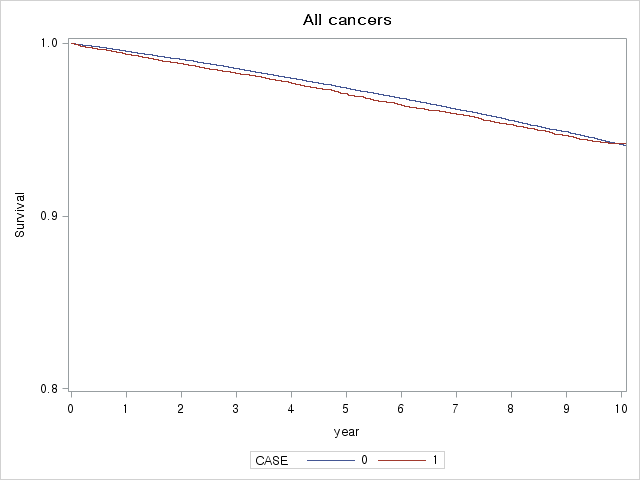

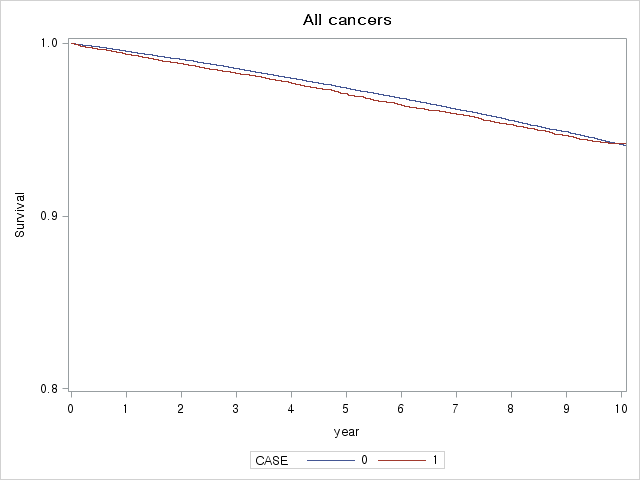

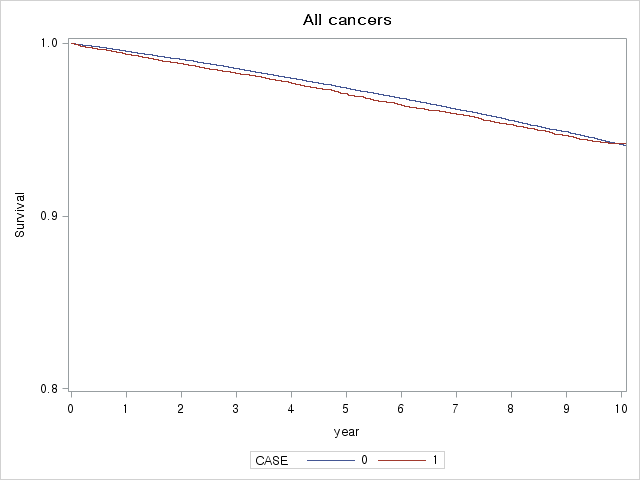


MHT users

Non-users


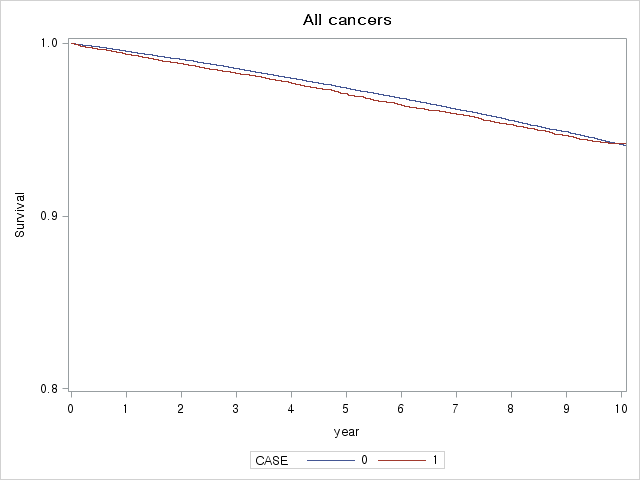


(D) Colorectal cancer


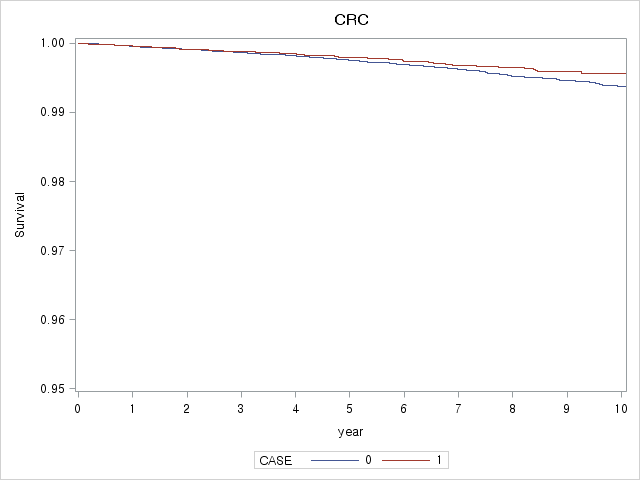


*p* for log-rank test = 0.0517


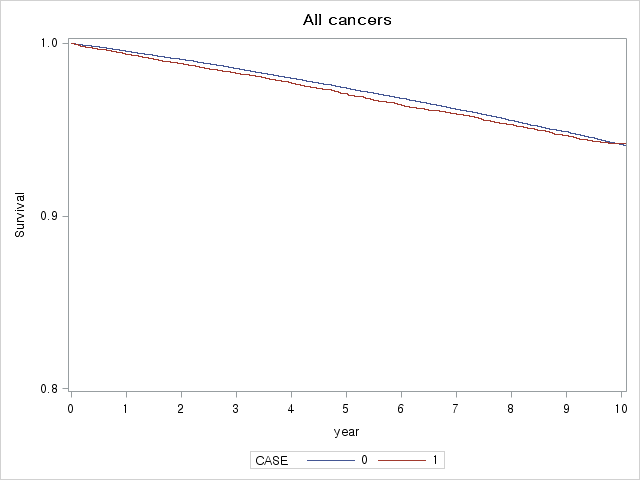

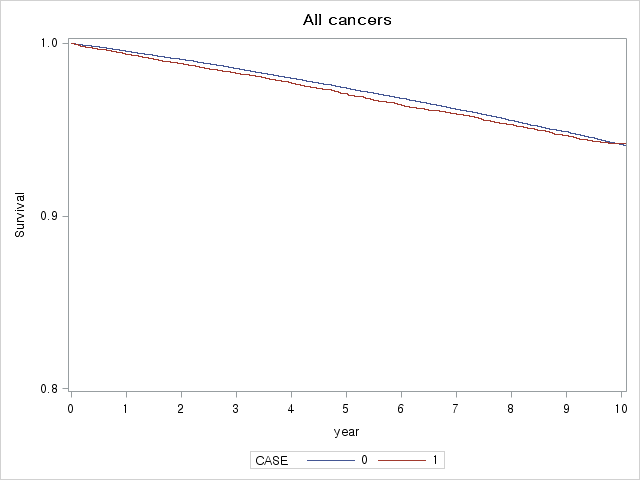

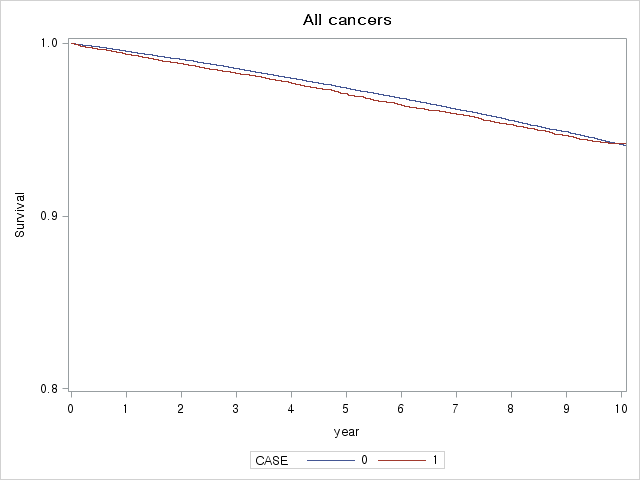


MHT users

Non-users


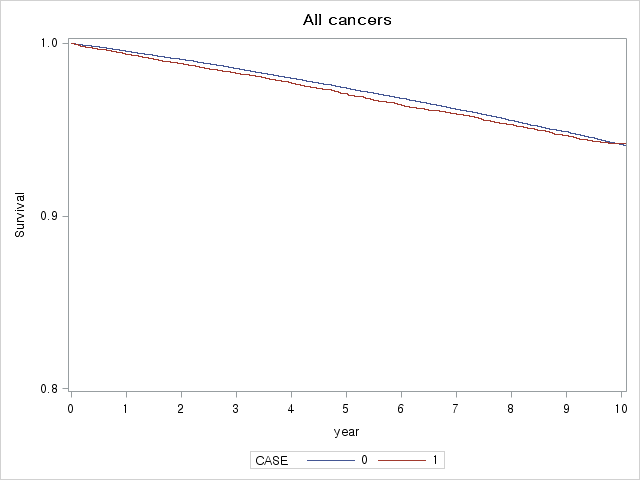


(E) Hepatobiliary cancer


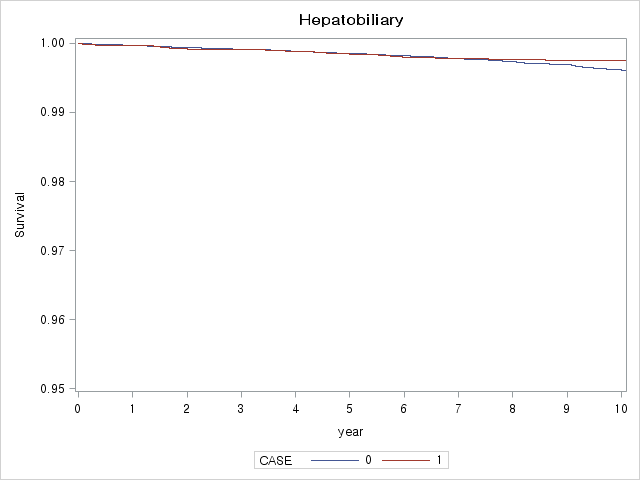


*p* for log-rank test = 0.2872


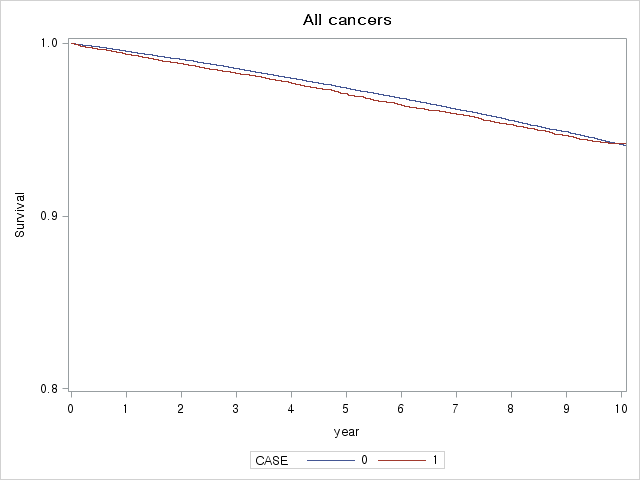

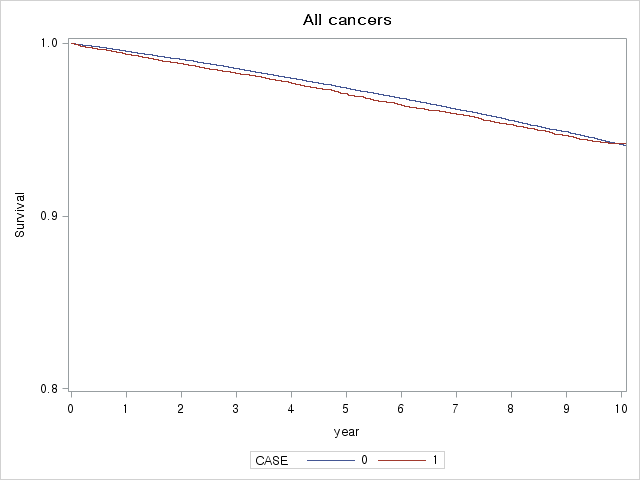

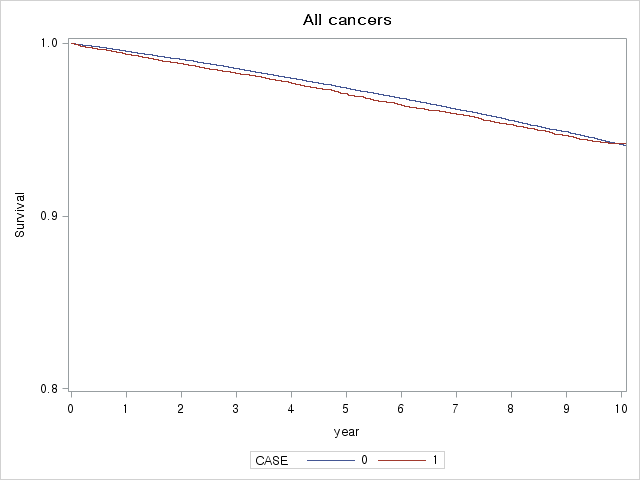


MHT users

Non-users


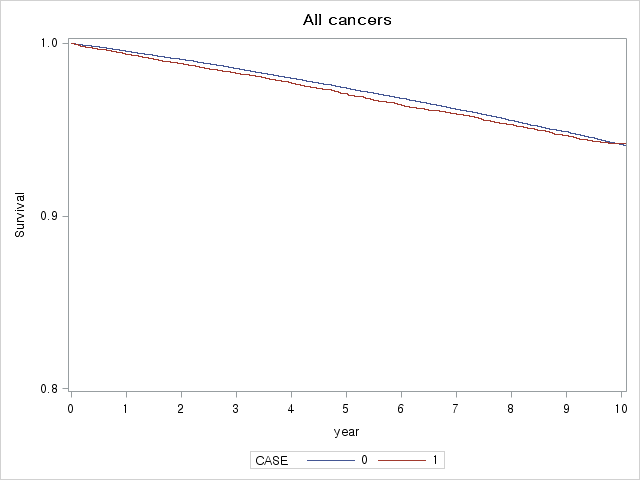


(F) Pancreatic cancer


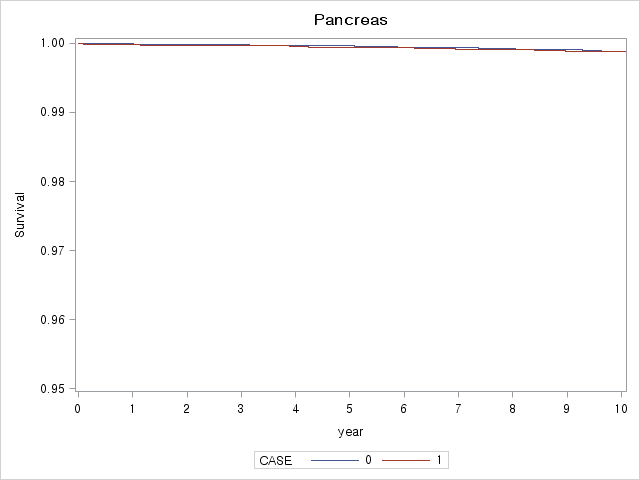


*p* for log-rank test = 0.5867


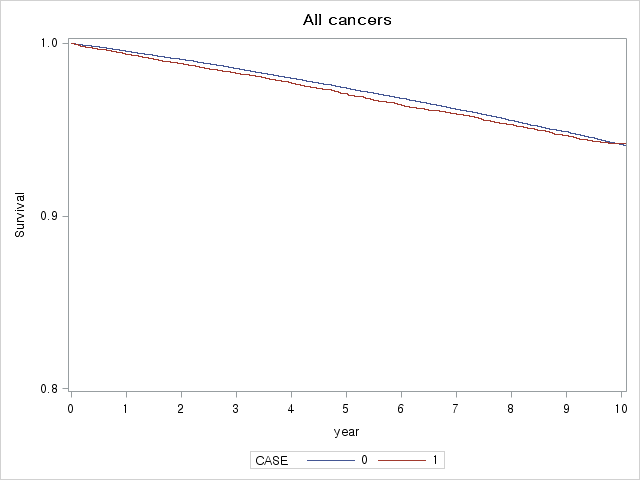

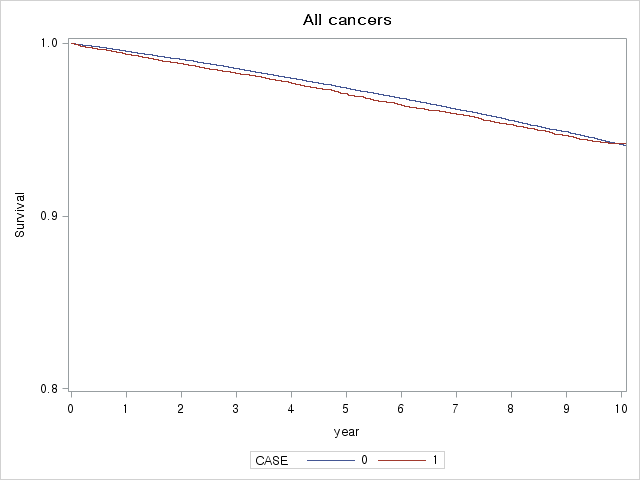

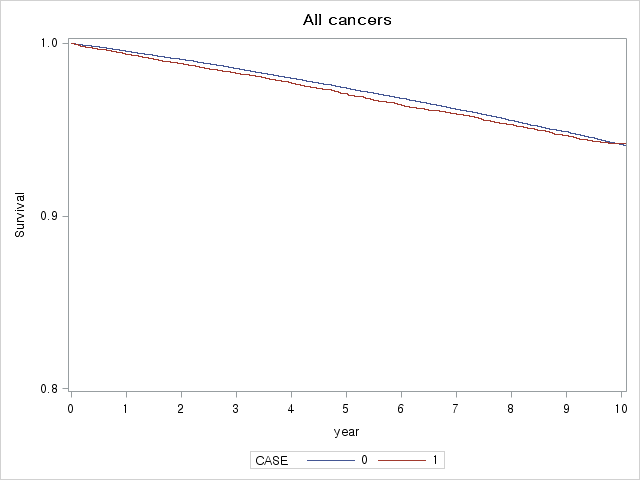


MHT users

Non-users


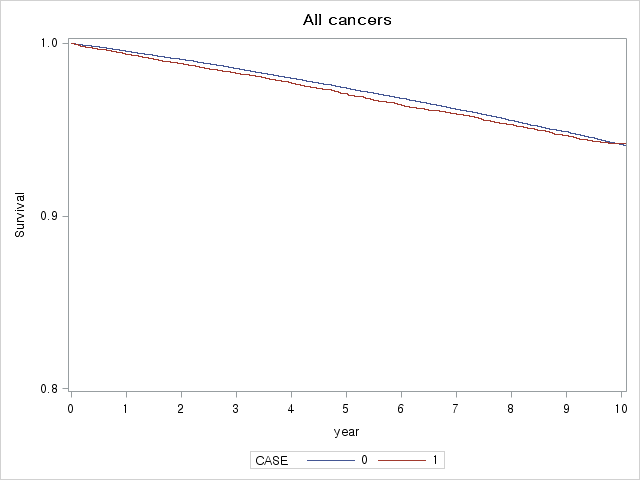

Supplement: Supplementary file 2 — Additional file 2. Figure S1. Kaplan-Meier survival curves for cancer incidence. Vertical lines indicate survival from diagnosis of cancer, and horizontal lines indicate observation years. [file 12876_2021_2021_MOESM2_ESM.docx]
