# Supplementary material for: The effect of menopausal hormone therapy on gastrointestinal cancer risk and mortality in South Korea: a population-based cohort study
Source: BMC Gastroenterol. 2021 Nov 23;21:440. doi: 10.1186/s12876-021-02021-y (PMC8609757; doi:10.1186/s12876-021-02021-y)
Supplement: Supplementary file 3 — Additional file 3. Table S2. Hazard ratios for development of cancers: subgroup analyses according to MHT regimens. [file 12876_2021_2021_MOESM3_ESM.docx]

**The effect of menopausal hormone therapy on gastrointestinal cancer risk and mortality in South Korea: a population-based cohort study**

**Table S2** Hazard ratios for development of cancers: subgroup analyses according to MHT regimens

|  |  | Any cancer | | |  | GI cancer | | |  | | Gastric cancer | | |
| --- | --- | --- | --- | --- | --- | --- | --- | --- | --- | --- | --- | --- | --- |
|  | n (%) | HR | 95% CI | *P* |  | HR | 95% CI | *P* | |  | HR | 95% CI | *P* |
| MHT type |  |  |  |  |  |  |  |  | |  |  |  |  |
| Estradiol^a^ | 9,125 (40.42) | 1.064 | 0.944, 1.199 | 0.3082 |  | 0.823 | 0.631, 1.072 | 0.1485 | |  | 0.742 | 0.473, 1.164 | 0.1943 |
| Conjugated estrogen^a^ | 2,147 (9.51) | 1.154 | 0.953, 1.397 | 0.1417 |  | 0.984 | 0.675, 1.435 | 0.9343 | |  | 0.895 | 0.461, 1.738 | 0.7436 |
| Tibolone | 6,102 (27.03) | 1.093 | 0.958, 1.247 | 0.1844 |  | 0.817 | 0.627, 1.066 | 0.1364 | |  | 0.891 | 0.579, 1.371 | 0.5987 |
| Mixed | 5,203 (23.05) | 0.949 | 0.823, 1.095 | 0.4733 |  | 0.689 | 0.498, 0.952 | 0.0242 | |  | 0.669 | 0.392, 1.142 | 0.1405 |
| Combination^b^ |  |  |  |  |  |  |  |  | |  |  |  |  |
| Estrogen only regimen | 4,566 (20.22) | 1.258 | 1.092, 1.450 | 0.0015 |  | 1.037 | 0.772, 1.394 | 0.8076 | |  | 0.736 | 0.414, 1.307 | 0.2952 |
| Combination regimen | 6,575 (29.12) | 0.958 | 0.831, 1.105 | 0.5593 |  | 0.728 | 0.531, 0.998 | 0.0488 | |  | 0.821 | 0.505, 1.335 | 0.4265 |
| Mixed | 4,467 (19.79) | 0.892 | 0.759, 1.049 | 0.1662 |  | 0.722 | 0.508, 1.027 | 0.0700 | |  | 0.647 | 0.355, 1.179 | 0.1549 |

continued

|  |  | Colorectal cancer | | |  | Hepatobiliary cancer | | |  | | Pancreatic cancer | | |
| --- | --- | --- | --- | --- | --- | --- | --- | --- | --- | --- | --- | --- | --- |
|  | n (%) | HR | 95% CI | *P* |  | HR | 95% CI | *P* | |  | HR | 95% CI | *P* |
| MHT type |  |  |  |  |  |  |  |  | |  |  |  |  |
| Estradiol^a^ | 9,125 (40.42) | 0.754 | 0.475, 1.197 | 0.2312 |  | 1.023 | 0.596, 1.758 | 0.9797 | |  | 1.253 | 0.503, 3.119 | 0.6279 |
| Conjugated estrogen^a^ | 2,147 (9.51) | 0.926 | 0.477, 1.798 | 0.8198 |  | 0.915 | 0.405, 2.065 | 0.8302 | |  | 2.141 | 0.773, 5.931 | 0.1433 |
| Tibolone | 6,102 (27.03) | 0.716 | 0.446, 1.151 | 0.1680 |  | 0.838 | 0.478, 1.468 | 0.5361 | |  | 0.818 | 0.297, 2.252 | 0.6975 |
| Mixed | 5,203 (23.05) | 0.731 | 0.428, 1.248 | 0.2502 |  | 0.611 | 0.288, 0.299 | 0.2005 | |  | 0.998 | 0.312, 3.189 | 0.9967 |
| Combination^b^ |  |  |  |  |  |  |  |  | |  |  |  |  |
| Estrogen only regimen | 4,566 (20.22) | 1.094 | 0.673, 1.781 | 0.7162 |  | 1.070 | 0.567, 2.017 | 0.8350 | |  | 2.536 | 1.161, 5.541 | 0.0196 |
| Combination regimen | 6,575 (29.12) | 0.566 | 0.310, 1.031 | 0.0627 |  | 0.900 | 0.478, 1.697 | 0.7455 | |  | 0.641 | 0.157, 2.619 | 0.5353 |
| Mixed | 4,467 (19.79) | 0.776 | 0.436, 1.380 | 0.3873 |  | 0.668 | 0.297, 1.506 | 0.3307 | |  | 1.268 | 0.396, 4.055 | 0.6891 |

Other covariates (age group, income, region, Charlson comorbidity index, and year of study entry) were adjusted in each survival analysis.

CI, confidence interval; DDD, defined daily dose; GI, gastrointestinal; HR, hazard ratio; MHT, menopausal hormone therapy.

^a^Included combination regimens.

^b^Except for 6,969 persons taking tibolone.
