# Supplementary material for: The effect of menopausal hormone therapy on gastrointestinal cancer risk and mortality in South Korea: a population-based cohort study
Source: BMC Gastroenterol. 2021 Nov 23;21:440. doi: 10.1186/s12876-021-02021-y (PMC8609757; doi:10.1186/s12876-021-02021-y)
Supplement: Supplementary file 4 — Additional file 4. Table S3. Hazard ratiosa for development of cancers: subgroup analyses according to baseline characteristics. [file 12876_2021_2021_MOESM4_ESM.docx]

**The effect of menopausal hormone therapy on gastrointestinal cancer risk and mortality in South Korea: a population-based cohort study**

**Table S3** Hazard ratios^a^ for development of cancers: subgroup analyses according to baseline characteristics

|  | Any cancer | | |  | GI cancer | | |  | Gastric cancer | | |
| --- | --- | --- | --- | --- | --- | --- | --- | --- | --- | --- | --- |
|  | HR | 95% CI | *P* |  | HR | 95% CI | *P* |  | HR | 95% CI | *P* |
| Age at MHT |  |  |  |  |  |  |  |  |  |  |  |
| 40-49 | 0.996 | 0.882, 1.124 | 0.9440 |  | 0.948 | 0.709, 1.269 | 0.7214 |  | 1.121 | 0.736, 1.707 | 0.5948 |
| 50-59 | 1.030 | 0.919, 1.155 | 0.6081 |  | 0.657 | 0.509, 0.846 | 0.0011 |  | 0.654 | 0.427, 1.002 | 0.0511 |
| ≥60 | 1.224 | 1.030, 1.455 | 0.0217 |  | 0.923 | 0.699, 1.220 | 0.5740 |  | 0.671 | 0.384, 1.173 | 0.1617 |
| Income level |  |  |  |  |  |  |  |  |  |  |  |
| -30% (low) | 1.012 | 0.885, 1.156 | 0.8633 |  | 0.747 | 0.557, 1.000 | 0.0498 |  | 0.624 | 0.371, 1.050 | 0.0758 |
| 31-60% | 1.033 | 0.895, 1.191 | 0.6581 |  | 0.763 | 0.569, 1.023 | 0.0703 |  | 0.807 | 0.502, 1.295 | 0.3739 |
| 61-90% | 1.009 | 0.865, 1.177 | 0.9097 |  | 0.785 | 0.564, 1.093 | 0.1511 |  | 0.785 | 0.454, 1.356 | 0.3851 |
| +91% (high) | 1.249 | 1.044, 1.495 | 0.0150 |  | 1.048 | 0.734, 1.496 | 0.7972 |  | 1.071 | 0.599, 1.915 | 0.8169 |
| Region |  |  |  |  |  |  |  |  |  |  |  |
| Metropolitan | 1.064 | 0.958, 1.182 | 0.2470 |  | 0.728 | 0.580, 0.915 | 0.0065 |  | 0.767 | 0.528, 1.115 | 0.1648 |
| Others | 1.040 | 0.935, 1.157 | 0.4717 |  | 0.896 | 0.722, 1.113 | 0.3214 |  | 0.808 | 0.559, 1.168 | 0.2569 |
| CCI |  |  |  |  |  |  |  |  |  |  |  |
| 1 | 0.918 | 0.793, 1.062 | 0.2501 |  | 0.832 | 0.583, 1.187 | 0.3105 |  | 1.023 | 0.622, 1.683 | 0.9283 |
| 2 | 1.075 | 0.948, 1.219 | 0.2574 |  | 0.706 | 0.529, 0.941 | 0.0174 |  | 0.704 | 0.444, 1.115 | 0.1348 |
| 3 | 0.977 | 0.806, 1.184 | 0.8106 |  | 0.734 | 0.512, 1.050 | 0.0907 |  | 0.710 | 0.376, 1.344 | 0.2934 |
| 4+ | 1.260 | 1.078, 1.472 | 0.0036 |  | 0.971 | 0.734, 1.286 | 0.8382 |  | 0.786 | 0.450, 1.372 | 0.3970 |

continued

|  | Colorectal cancer | | |  | Hepatobiliary cancer | | |  | Pancreatic cancer | | |
| --- | --- | --- | --- | --- | --- | --- | --- | --- | --- | --- | --- |
|  | HR | 95% CI | *P* |  | HR | 95% CI | *P* |  | HR | 95% CI | *P* |
| Age at MHT |  |  |  |  |  |  |  |  |  |  |  |
| 40-49 | 0.722 | 0.426, 1.225 | 0.2276 |  | 0.920 | 0.429, 1.971 | 0.8293 |  | 1.794 | 0.569, 5.659 | 0.3188 |
| 50-59 | 0.678 | 0.442, 1.041 | 0.0754 |  | 0.752 | 0.457, 1.237 | 0.2618 |  | 0.330 | 0.079, 1.385 | 0.1299 |
| ≥60 | 0.898 | 0.557, 1.448 | 0.6599 |  | 0.905 | 0.512, 1.600 | 0.7309 |  | 1.875 | 0.904, 3.889 | 0.0913 |
| Income level |  |  |  |  |  |  |  |  |  |  |  |
| -30% (low) | 0.667 | 0.395, 1.126 | 0.1297 |  | 0.920 | 0.520, 1.628 | 0.7748 |  | 1.192 | 0.447, 3.178 | 0.7249 |
| 31-60% | 0.625 | 0.371, 1.053 | 0.0777 |  | 0.777 | 0.411, 1.468 | 0.4369 |  | 1.789 | 0.641, 4.989 | 0.2664 |
| 61-90% | 0.858 | 0.495, 1.488 | 0.5863 |  | 0.644 | 0.292, 1.419 | 0.2748 |  | 1.060 | 0.359, 3.131 | 0.9160 |
| +91% (high) | 1.082 | 0.593, 1.973 | 0.7979 |  | 1.079 | 0.499, 2.332 | 0.8467 |  | 0.718 | 0.163, 3.165 | 0.6620 |
| Region |  |  |  |  |  |  |  |  |  |  |  |
| Metropolitan | 0.593 | 0.394, 0.892 | 0.0120 |  | 0.808 | 0.491, 1.331 | 0.4035 |  | 1.182 | 0.569, 2.453 | 0.6542 |
| Others | 0.958 | 0.663, 1.384 | 0.8182 |  | 0.883 | 0.560, 1.391 | 0.5914 |  | 1.157 | 0.508, 2.633 | 0.7284 |
| CCI |  |  |  |  |  |  |  |  |  |  |  |
| 1 | 0.631 | 0.326, 1.223 | 0.1726 |  | 0.803 | 0.310, 2.080 | 0.6513 |  | 1.178 | 0.250, 5.548 | 0.8362 |
| 2 | 0.852 | 0.539, 1.346 | 0.4921 |  | 0.714 | 0.378, 1.351 | 0.3012 |  | NA | - | 0.9874 |
| 3 | 0.688 | 0.374, 1.268 | 0.2308 |  | 0.422 | 0.168, 1.059 | 0.0662 |  | 3.061 | 1.162, 8.063 | 0.0235 |
| 4+ | 0.766 | 0.455, 1.291 | 0.3176 |  | 1.280 | 0.778, 2.104 | 0.3310 |  | 1.418 | 0.618, 3.252 | 0.4093 |

Other covariates were adjusted in each survival analysis.

CCI, Charlson comorbidity index; CI, confidence interval; GI, gastrointestinal; HR, hazard ratio; MHT, menopausal hormone therapy.

^a^Hazard ratios for cancer development in MHT users compared to non-users.
