# Supplementary material for: The effect of menopausal hormone therapy on gastrointestinal cancer risk and mortality in South Korea: a population-based cohort study
Source: BMC Gastroenterol. 2021 Nov 23;21:440. doi: 10.1186/s12876-021-02021-y (PMC8609757; doi:10.1186/s12876-021-02021-y)
Supplement: Supplementary file 5 — Additional file 5. Figure S2. Kaplan-Meier survival curves for mortality. Vertical lines indicate survival from death, and horizontal lines indicate observation years. [file 12876_2021_2021_MOESM5_ESM.docx]

**The effect of menopausal hormone therapy on gastrointestinal cancer risk and mortality in South Korea: a population-based cohort study**

**Figure S2**. Kaplan-Meier survival curves for mortality. Vertical lines indicate survival from death, and horizontal lines indicate observation years.

(A) All-cause mortality


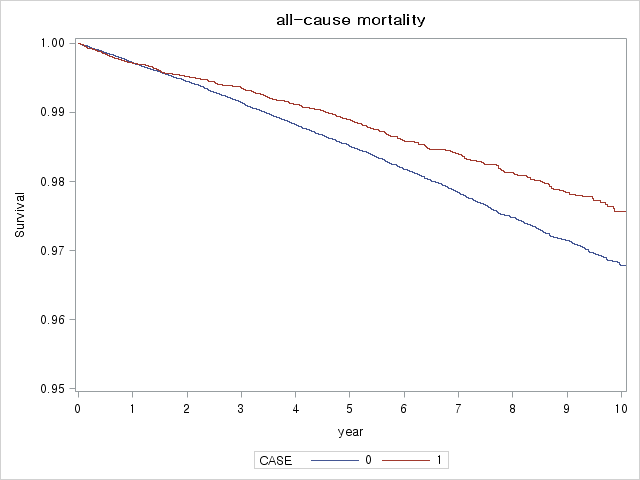


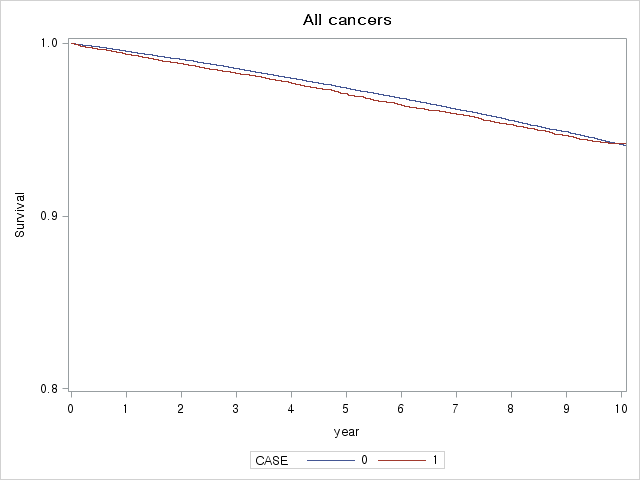

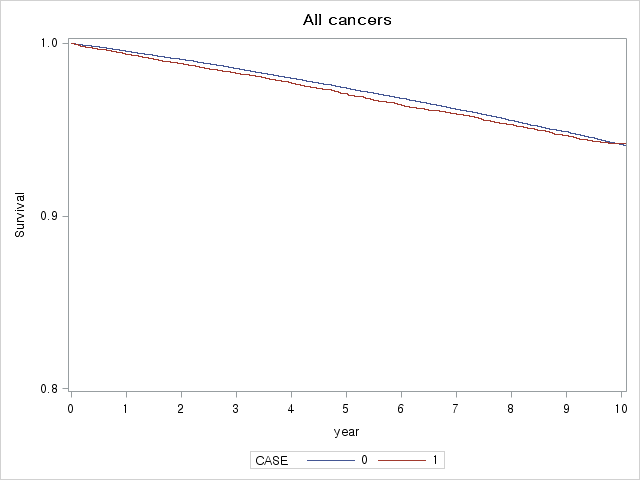

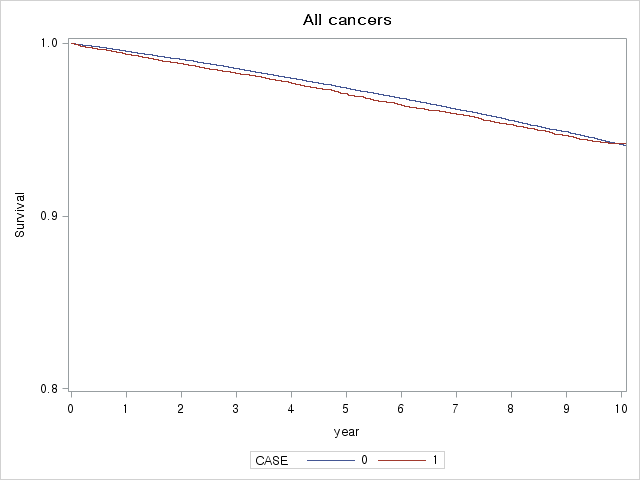


MHT users

Non-users


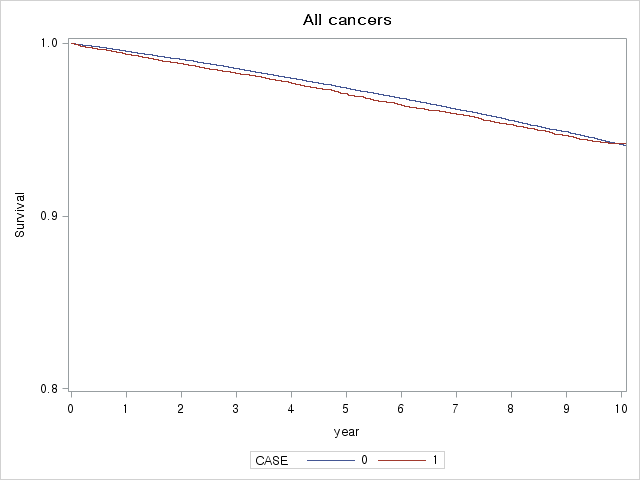


*p* for log-rank test <.0001

(B) Cancer-related mortality


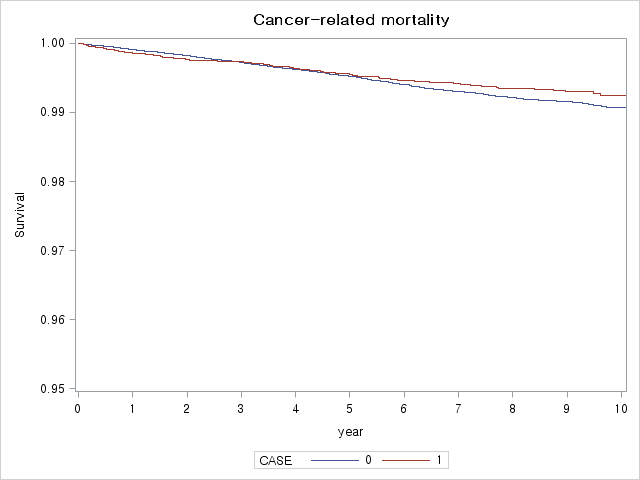


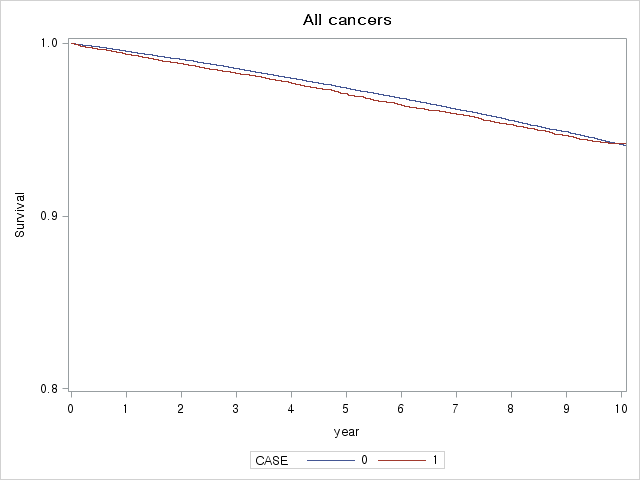

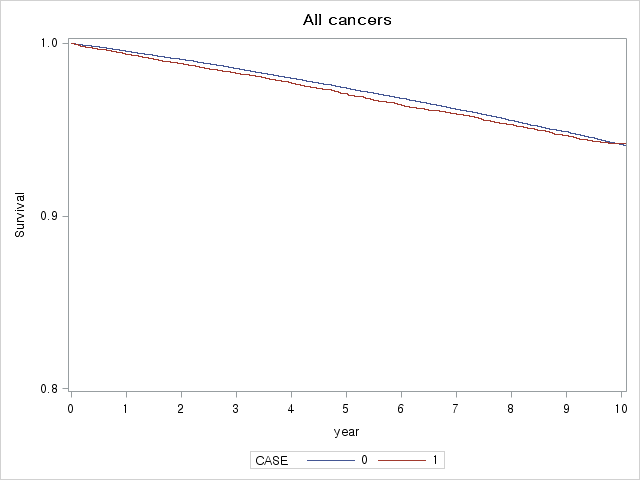

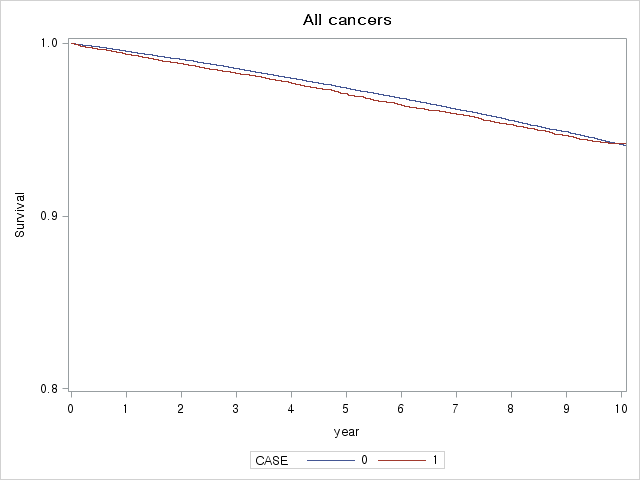


MHT users

Non-users


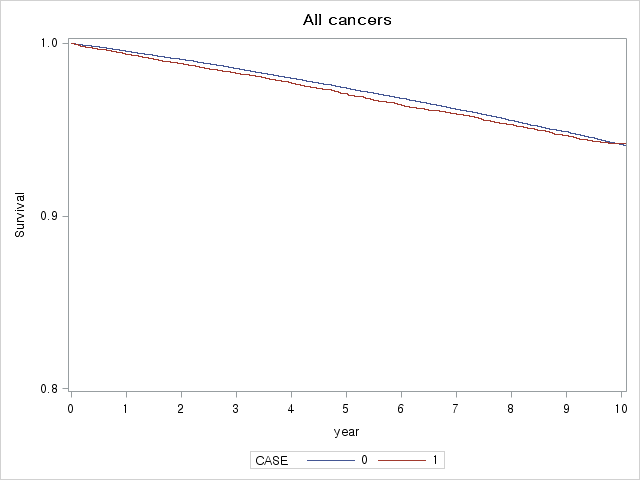


*p* for log-rank test = 0.1377

(C) Gastrointestinal cancer mortality


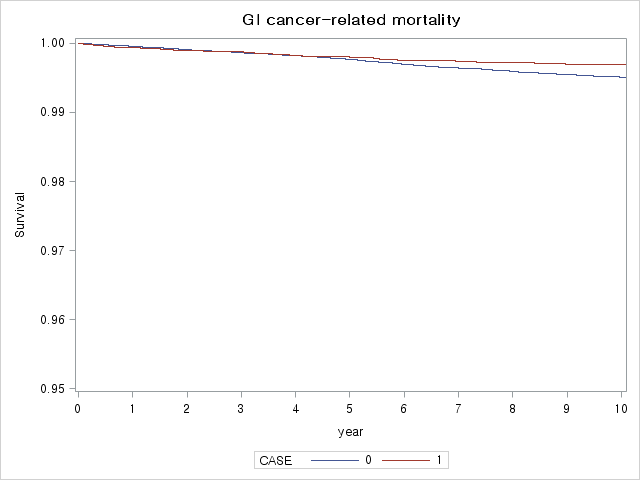


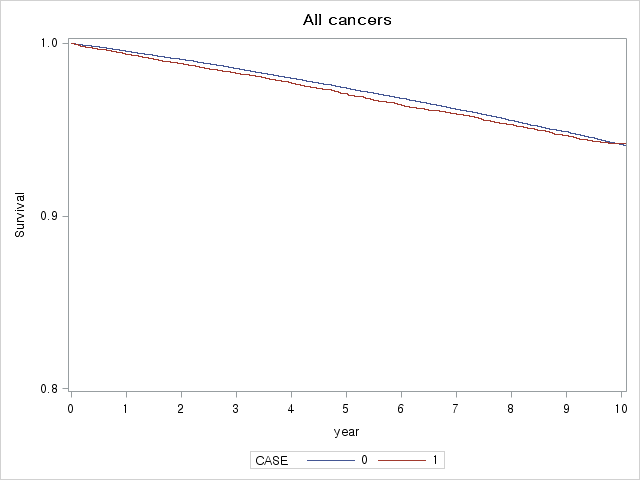

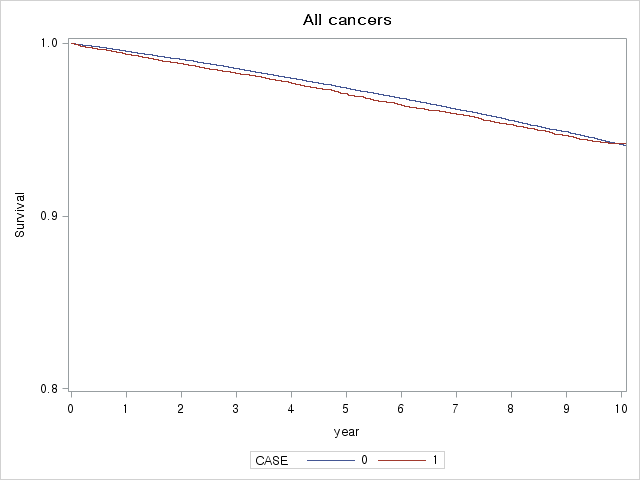

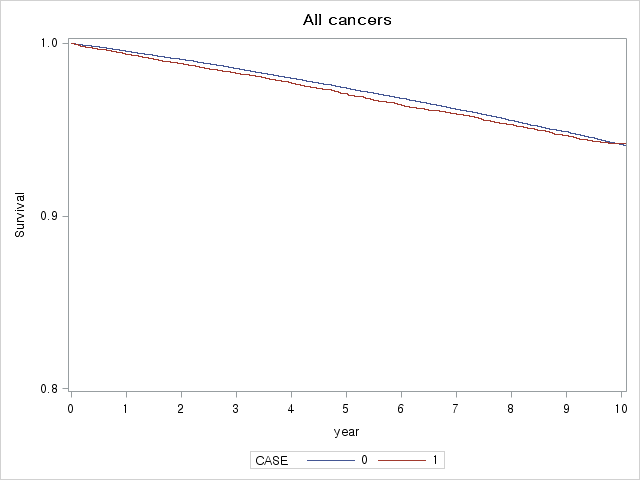


MHT users

Non-users


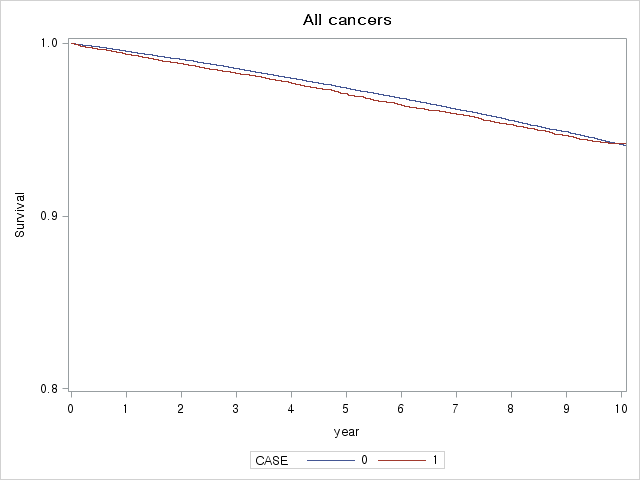


*p* for log-rank test = 0.0377
