# Supplementary material for: The effect of menopausal hormone therapy on gastrointestinal cancer risk and mortality in South Korea: a population-based cohort study
Source: BMC Gastroenterol. 2021 Nov 23;21:440. doi: 10.1186/s12876-021-02021-y (PMC8609757; doi:10.1186/s12876-021-02021-y)

**The effect of menopausal hormone therapy on gastrointestinal cancer risk and mortality in South Korea: a population-based cohort study**

**Figure S3**. Kaplan-Meier survival curves: dose-response relationship. Vertical lines

indicate survival from diagnosis of cancer, and horizontal lines indicate observation years. 5 groups were based on DDD of MHT (1, ≤100; 2, 100~300; 3, 300~600; 4, ≥600; and no MHT). DDD, defined daily dose; MHT, menopausal hormone therapy

(A) Gastric cancer incidence


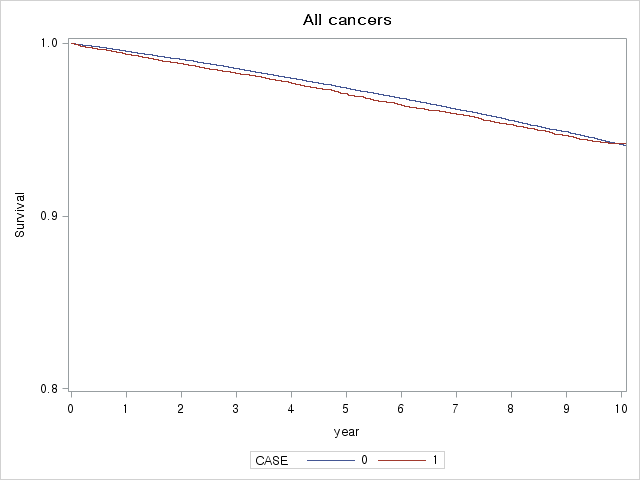

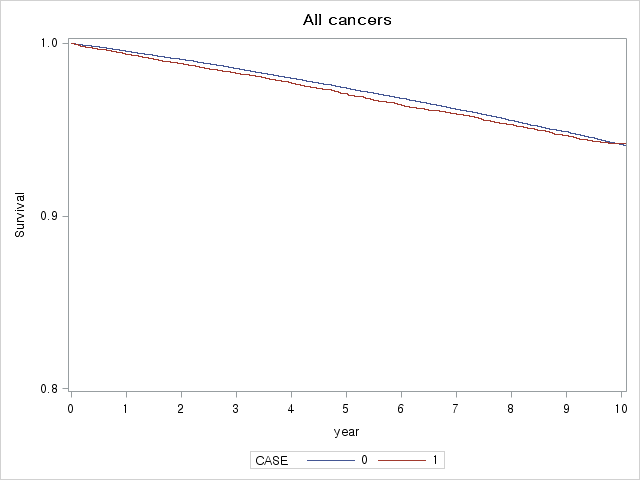


*p* for log-rank test = 0.2146


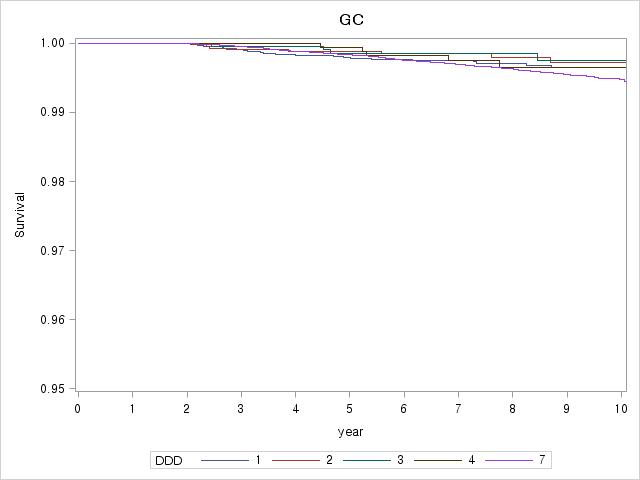


no MHT

DDD
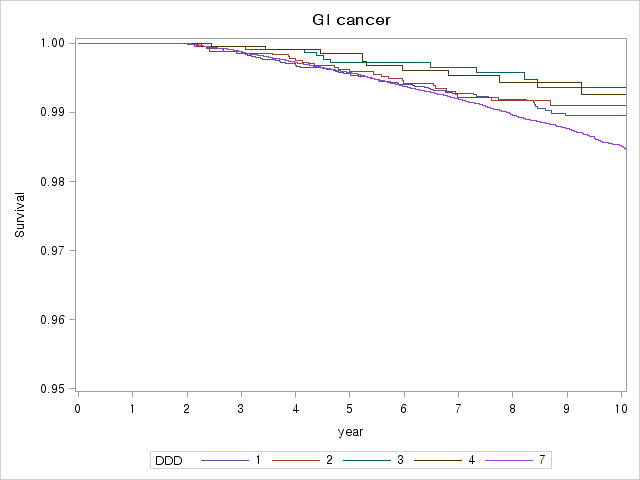

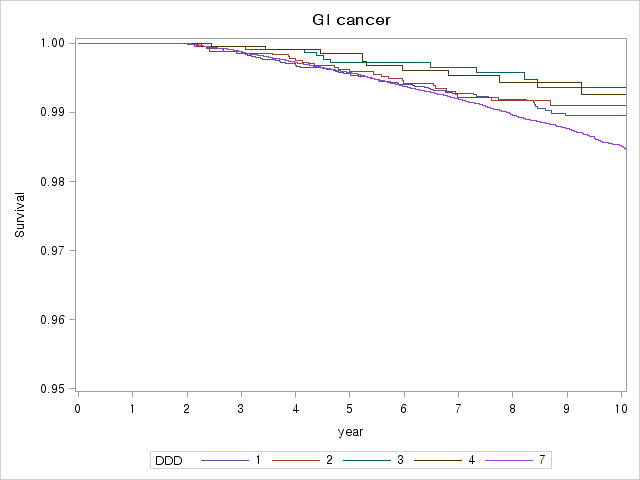


(B) Colorectal cancer incidence


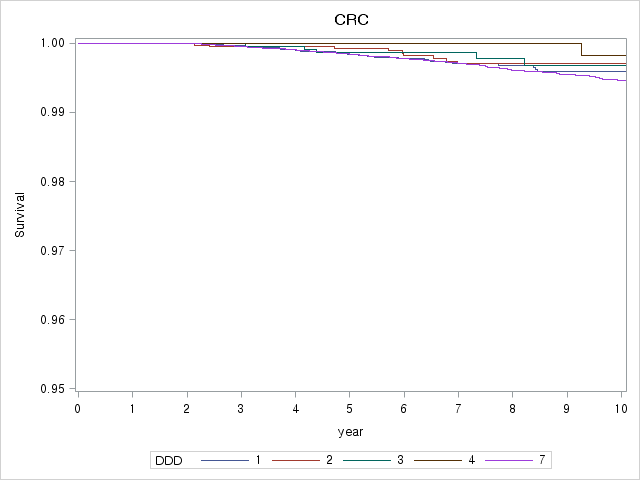


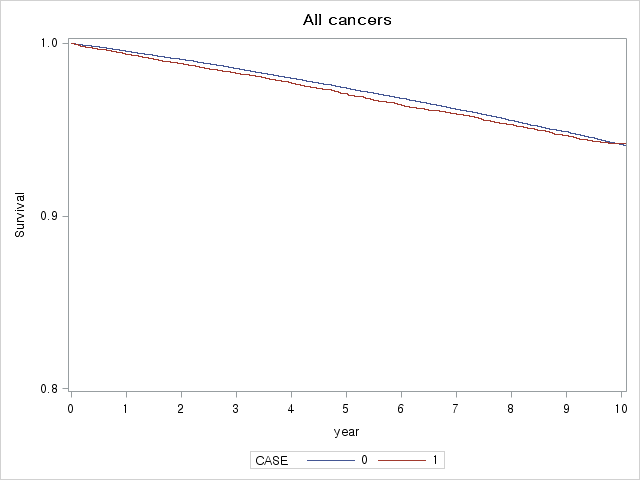

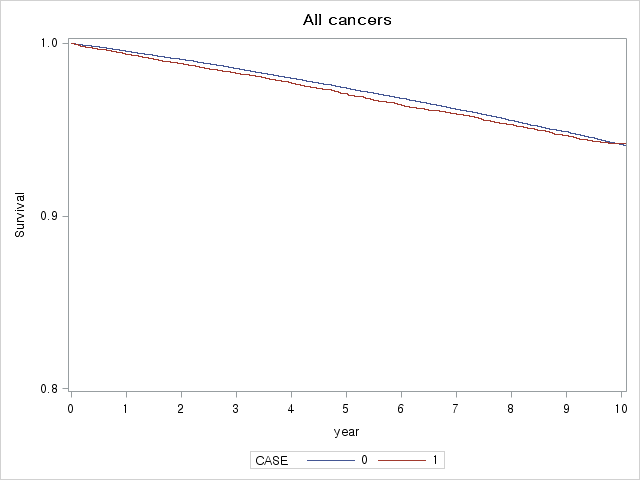


*p* for log-rank test = 0.0896

no MHT

DDD
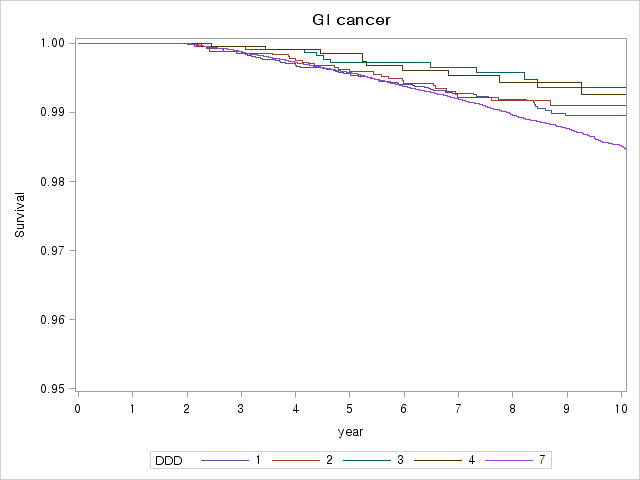

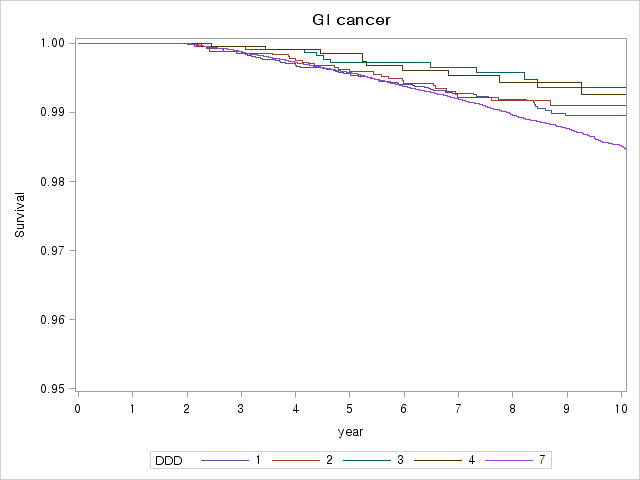

Supplement: Supplementary file 6 — Additional file 6. Figure S3. Kaplan-Meier survival curves: dose-response relationship. Vertical linesindicate survival from diagnosis of cancer, and horizontal lines indicate observation years. 5 groups were based on DDD of MHT (1, ≤ 100; 2, 100~300; 3, 300~600; 4, ≥ 600; and no MHT). DDD, defined daily dose; MHT, menopausal hormone therapy. [file 12876_2021_2021_MOESM6_ESM.docx]
