# Supplementary material for: The effect of menopausal hormone therapy on gastrointestinal cancer risk and mortality in South Korea: a population-based cohort study
Source: BMC Gastroenterol. 2021 Nov 23;21:440. doi: 10.1186/s12876-021-02021-y (PMC8609757; doi:10.1186/s12876-021-02021-y)
Supplement: Supplementary file 7 — Additional file 7. Table S4. Sensitivity analyses of cancer incidence using Landmark data set (n = 116,091). [file 12876_2021_2021_MOESM7_ESM.docx]

**The effect of menopausal hormone therapy on gastrointestinal cancer risk and mortality in South Korea: a population-based cohort study**

**Table S4** Sensitivity analyses of cancer incidence using Landmark data set (n = 116,091)

| Outcome variables | MHT users | |  | Non-users | | HR^b^ | 95% CI | *P* |
| --- | --- | --- | --- | --- | --- | --- | --- | --- |
|  | n | rate^a^ |  | n | rate^a^ |  |  |  |
| Any cancer | 591 | 0.422 |  | 2,960 | 0.428 | 0.981 | 0.898, 1.072 | 0.6792 |
| GI cancer | 120 | 0.086 |  | 836 | 0.121 | 0.703 | 0.580, 0.852 | 0.0003 |
| Esophageal | 0 | 0.000 |  | 4 | 0.001 | - | - | 0.9977 |
| Gastric | 43 | 0.031 |  | 303 | 0.044 | 0.684 | 0.497, 0.943 | 0.0202 |
| Colorectal | 42 | 0.030 |  | 297 | 0.043 | 0.693 | 0.502, 0.958 | 0.0266 |
| Hepatobiliary | 24 | 0.017 |  | 174 | 0.025 | 0.689 | 0.449, 1.057 | 0.0883 |
| Pancreatic | 10 | 0.007 |  | 55 | 0.008 | 0.89 | 0.453, 1.748 | 0.7341 |

Other covariates (age group, income, region, Charlson comorbidity index, and year of study entry) were adjusted in each survival analysis.

CI, confidence interval; GI, gastrointestinal; HR, hazard ratio; MHT, menopausal hormone therapy.

^a^Incidence rates per 100,000 person-years.

^b^Adjusted hazard ratios for cancer incidence in MHT users compared to non-users.
